# Supplementary material for: Health economic evaluation of strategies to eliminate gambiense human African trypanosomiasis in the Mandoul disease focus of Chad
Source: PLoS Negl Trop Dis. 2023 Jul 27;17(7):e0011396. doi: 10.1371/journal.pntd.0011396 (PMC10409297; doi:10.1371/journal.pntd.0011396)
Supplement: S1 Text — Fig A: Remaining gHAT foci in Chad. All remaining gHAT foci in Chad are located in the Southern region of the country. The exact extent of the area of transmission for Mandoul is hard to precisely define. The Mandoul focus was determined by geolocating all the gHAT cases indicated as living in Mandoul in the WHO HAT Atlas. Reprinted from Rock et al. [21] under a CC-BY license. Table E: Summary of passive screening in fixed health facilities. Table D: Summary of active screening activities by mini-mobile teams in motorcycles. Table C: Summary of active screening activities by traditional teams in trucks. Note: some of these cases might not be parasitologically confirmed. S1.2 Text. Additional method details covering the transmission model, strategy components, treatment model and health outcomes denominated as disability-adjusted life-years (DALYs). S1.2.3 Text. Additional method details about passive detection and unreported deaths. Fig C: The transmission and treatment models The transmission model, depicting a Susceptible-Exposed-Infected-Recovered-Susceptible (SEIRS) model, which represents the progression of disease among low-risk humans (blue compartments), tsetse (purple compartment) and high-risk humans (red compartments), and the transmission of disease between the three groups. B) The probability tree representing treatment outcomes. Before 2020, a smaller tree was used constituting only the NECT and pentamidine branches for stage 1 and 2 disease, respectively, as fexinidazole was unavailable. Reproduced under CC-BY from Rock et. al [21] (part A) and Antillon et. al [67] (part B). S1.4 Text. Additional method details covering the treatment model. S1.3 Text. Additional method details covering the strategy components. Table F: Parameters for treatment eligibility. Table G: Eligibility for treatment. Fig E: New 2000–2019 ensemble fit (incorporates 3.15% population growth). The top row shows the number of people screened annually in the Mandoul focus from 2000– [file pntd.0011396.s001.pdf]

Supporting Information

Health economic evaluations of strategies to eliminate *gambiense* human African trypanosomiasis in the Mandoul disease focus of Chad

S1. Supplementary Methods

Contents

S1.1 Locations . . . S1.3
S1.2 Transmission model . . . S1.4
S1.2.1 Overview . . . S1.4
S1.2.2 Re-fit with population growth and support for different model variants . . . S1.7
S1.2.3 Passive detection and unreported deaths . . . S1.11
S1.3 Strategy components . . . S1.15
S1.4 Treatment model . . . S1.16
S1.4.1 Health outcomes denominated as disability-adjusted life-years (DALYs) . . . S1.17
S1.5 Cost functions . . . S1.17
S1.5.1 Cost functions: active screening . . . S1.18
S1.5.2 Cost functions: passive screening (screening at fixed health posts) . . . S1.24
S1.5.3 Cost functions: vector control . . . S1.30
S1.5.4 Cost functions: treatment . . . S1.30
References for SI Text 1 . . . S1.32

List of Figures

A Remaining gHAT foci in Chad. . . . S1.3
B Decision trees . . . S1.5
C Transmission and treatment models . . . S1.6
D Priors and posteriors for ensemble models . . . S1.9
E New 2000–2019 ensemble fit (incorporates 3.15% population growth) . . . S1.10
F New counterfactual strategy predictions 2014–2030 . . . S1.11
G Modelled progression routes for humans once infectious . . . S1.12
H Passive detection rates over time . . . S1.13
I Comparison of detected and undetected infections generated by the model and their relationship to active screening intensity . . . S1.14

List of Tables

A Different model structures under consideration and their relative DIC scores for the fitting to 2000–2019 data. . . . S1.7
B Ensemble posteriors of fitted parameters . . . S1.8
C Summary of active screening activities by traditional teams . . . S1.15
D Summary of active screening activities by mini-mobile teams . . . S1.16
E Summary of passive screening in fixed health facilities . . . S1.16
F Parameters for treatment eligibility. . . . S1.17
G Eligibility for treatment . . . S1.17
H Treatments and outcomes distributions for stage 1 and 2 patients . . . S1.18
I Active screening: cost function . . . S1.19
J Components of active screening costs . . . S1.20
K Cost breakdown for active screening activities . . . S1.20
L Passive screening: cost function . . . S1.25
M Components of passive screening costs . . . S1.26
N Cost breakdown for passive screening activities . . . S1.27
O Treatment: cost function . . . S1.30

P Parameters for treatment costs S1.31
Q Cost per person for different gHAT treatments. Because these are costs averaged over all patients and SAEs are rare, the average cost per patient for SAE is low. S1.31

S1.1 Locations

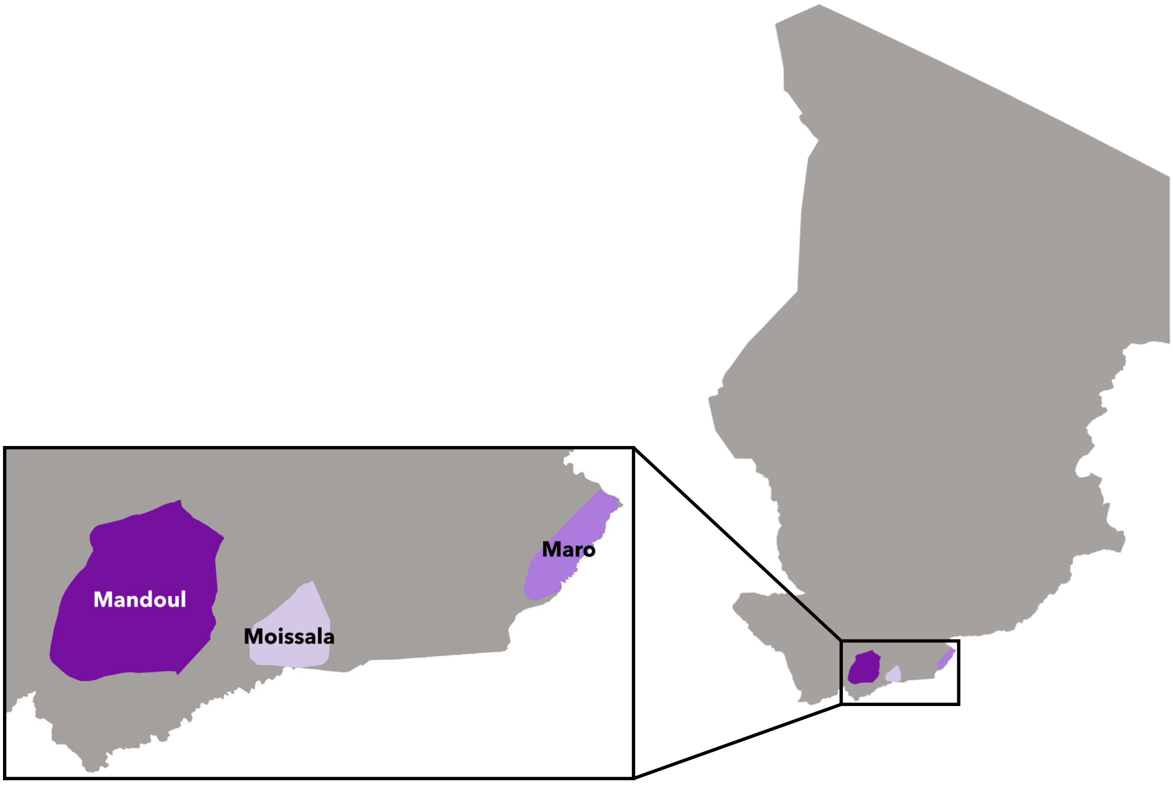

Figure A: Remaining gHAT foci in Chad. All remaining gHAT foci in Chad are located in the Southern region of the country. The exact extent of the area of transmission for Mandoul is hard to precisely define. The Mandoul focus was determined by geolocating all the gHAT cases indicated as living in Mandoul in the WHO HAT Atlas. Reprinted from Rock et al. [1] under a CC-BY license.

S1.2 Transmission model

S1.2.1 Overview

We took the deterministic transmission models by Rock et al. [1], and performed an updated fit to epidemiological data from the gHAT focus centered around Mandoul, Chad. The updated fit used identical data and fitting methods to that presented before [1] however in this present study we accounted for the 3.15% annual population growth believed to have occurred in the Mandoul focus. The models used are a derivative of the 'Warwick' gHAT models developed by Rock et al. [3] for gHAT transmission in the Democratic Republic of Congo and adapted for the Chadian context by Mahamat et al. [4] before the latest update [1]. The models used here were described in detail in Rock et al. [1] and include heterogeneity in people's exposure to tsetse bites, some systematic non-participation in active screening (AS), and the possibility of non-human animal transmission (see Fig C). A Markov chain Monte Carlo (MCMC) approach was used to fit the model to staged, active, and passive case data (2000–2019) from the WHO HAT Atlas and PNLTHA-Chad. The model variants used in the present study were selected based on statistical support for each based on Mandoul's human case data and weighted accordingly – this created an "ensemble" model which captures some structural uncertainty in gHAT epidemiology in Mandoul. The ensemble model – further described in Section S1.2.2 in this supplement. How the transmission model calculates detected cases (active and passive) and undetected deaths each year is then described in Section S1.2.3 in this supplement.

We used the transmission model to project the impact of four strategies for our retrospective analysis: the status quo practice before 2014 of average AS, passive screening (PS), and no vector control (VC), our referent case, and three other strategies made up of combinations of these interventions (and described in the main text and in Section S1.3) in this supplement. Likewise, prospective strategies described in the main text were simulated using this transmission model. While our primary outcome in the present study is the disability-adjusted life-year (DALY), we will also consider each strategy's capacity to reach the WHO's 2030 goal for gHAT: to stop transmission by 2030. In the deterministic model, we use a proxy threshold of < 1 new infection per year to approximate when EoT occurs.

We estimated the new cases and undetected deaths by triangulating various pieces of information which are included in simulations: 1) variable screening and corresponding case reporting – years with less screening typically result in fewer case detections and more people dying undetected and 2) the ratio of stage 1 versus stage 2 cases identified – more stage 1 cases indicate more ongoing new transmission and more stage 2 cases indicate cases infected longer before. We also note that the stage ratio is expected to be skewed to early detection with AS which enables even people with mild or no symptoms to be identified and treated, whereas passive detection typically finds more people with stage 2 disease. From these ratios, we can use the model fitting procedure to estimate the number of people who stop having stage 2 infection due to unreported deaths rather than those reported in the case counts. The parameter determining the proportion of infections not picked up by passive screening that will go on to be reported rather than die (u) is one of the model parameters which is estimated through fitting to the case data. This is further discussed in Section S1.2.3 in this supplement.

Sensitivity analysis looked at the economic case of these interventions in the hypothetical scenario that fexinidazole had been available from 2014, as well as if the strategies remain a good-value-for-money with longer and shorter time horizons and with no discount rate. Structural uncertainty in the epidemiological model was taken into account via the ensemble model, and parameter uncertainty was incorporated by both the transmission model parameter estimation using the MCMC approach and using the net benefits framework with uncertainty in costs. Finally, value-of-information analysis was performed to assess the most important sources of uncertainty.

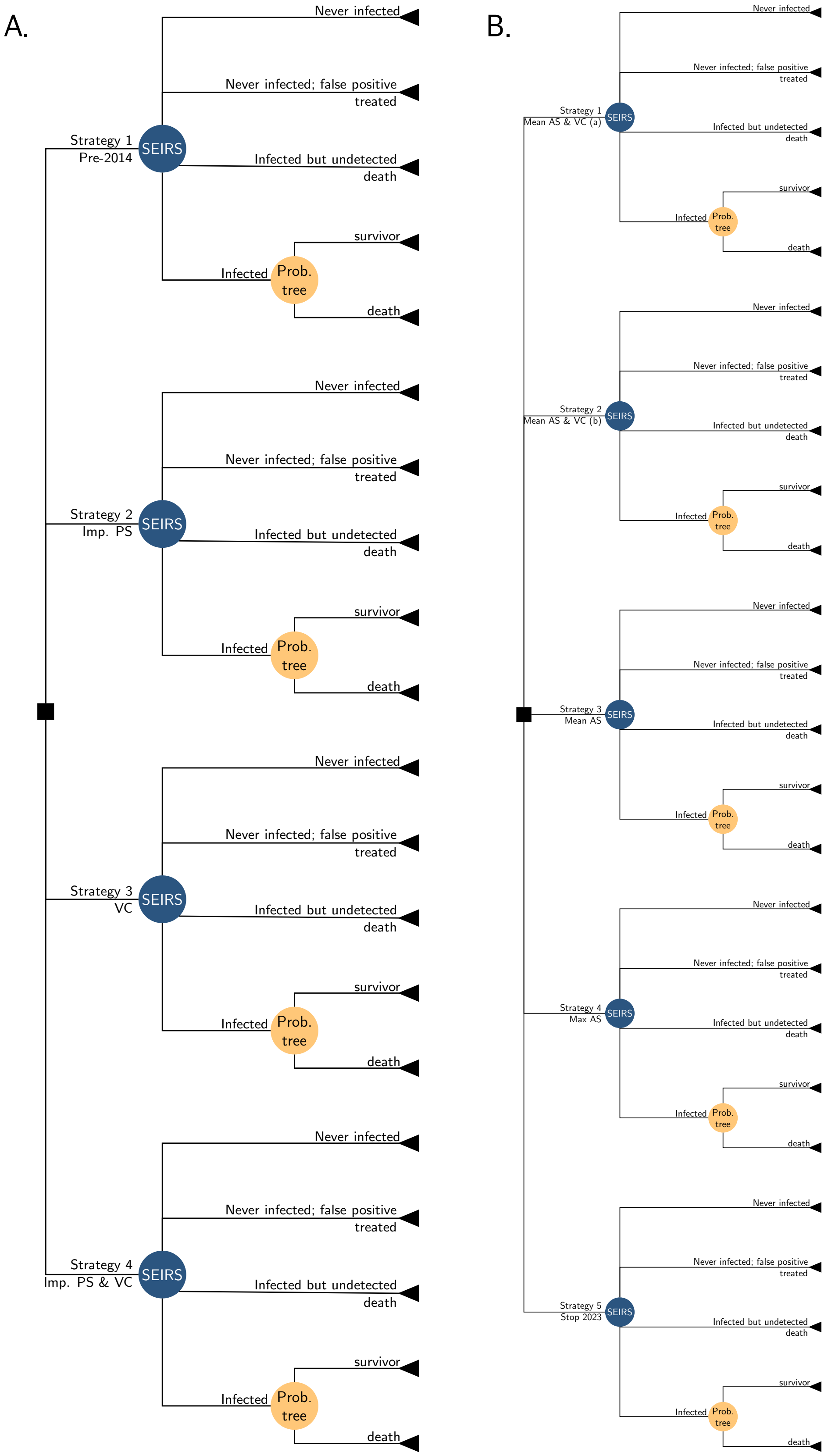

Figure B: A) Decision tree among four strategies for the retrospective analysis. B) A fifth branch is added for the prospective analysis, as there were 5 strategies to compare. For each strategy, the top two resulting branches of the model come from the transmission model only, and the bottom two resulting branches come from the treatment tree, which is fed outputs of detected cases from the transmission tree.

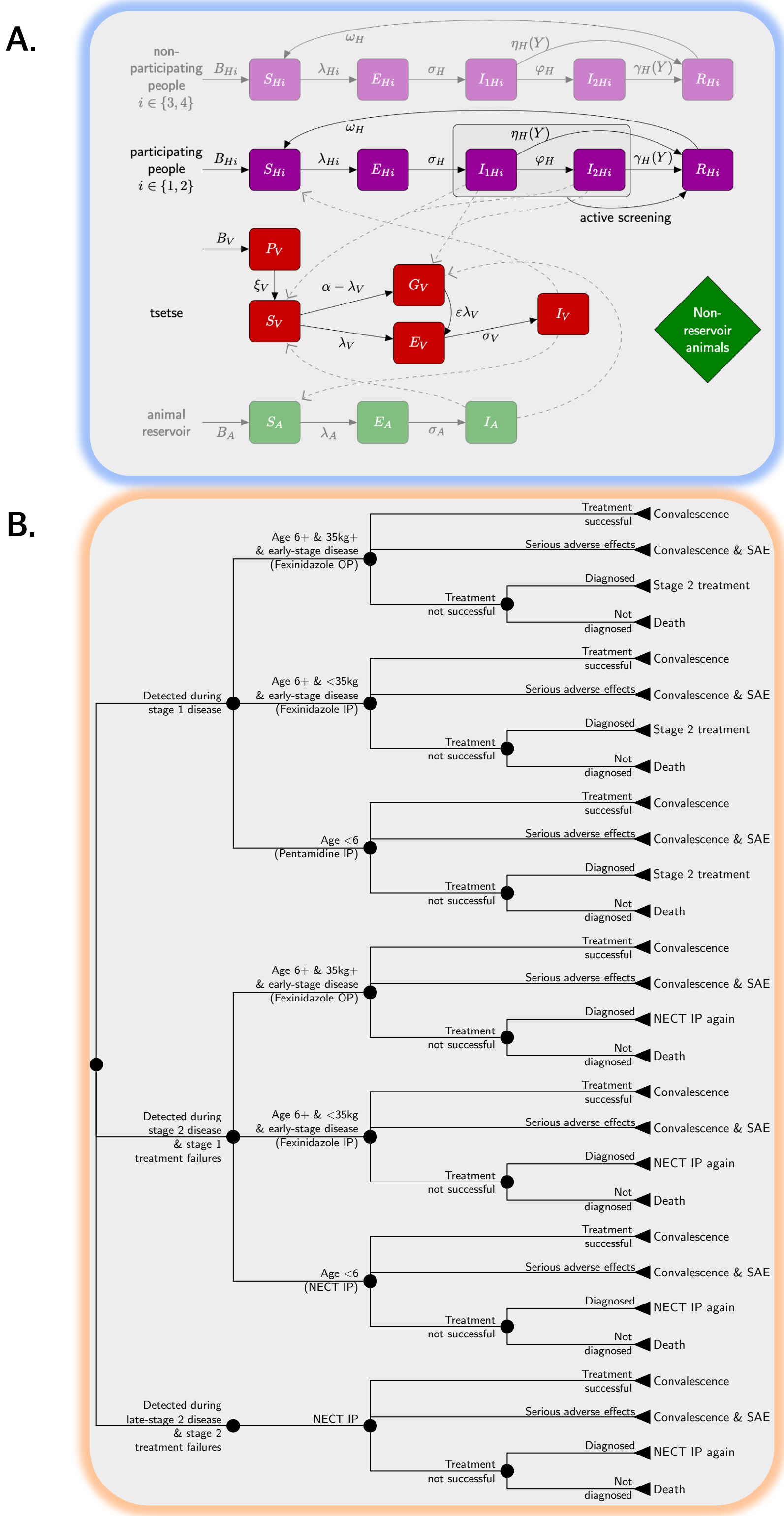

Figure C: A) The transmission model, depicting a Susceptible-Exposed-Infected-Recovered-Susceptible (SEIRS) model, which represents the progression of disease among low-risk humans (blue compartments), tsetse (purple compartment) and high-risk humans (red compartments), and the transmission of disease between the three groups. B) The probability tree representing treatment outcomes. Before 2020, a smaller tree was used constituting only the NECT and pentamidine branches for stage 1 and 2 diseases, respectively, as fexinidazole was unavailable. Reproduced under CC-BY from *Rock et. al* [1] (part A) and *Antillon et. al* [2] (part B).

S1.2.2 Re-fit with population growth and support for different model variants

To examine which model variants were most supported through fitting to the longitudinal data, we used the deviance information criterion (DIC),

$$DIC = -2LL(\bar{\theta}) + 4Var(LL(\theta)) \quad (1)$$

which assigns a lower score to models with high posterior mean log-likelihood whilst penalising models with a larger number of parameters [5]. The relative likelihood of model i was computed using,

$$\text{Relative } DIC = \exp\left((DIC_{min} - DIC_i)/2\right) \quad (2)$$

and was used to compare models. This method was the same as used for previous model fitting and selection [4].

Model	Random participation		Non-participation		Animals	Relative DIC	
	Low-risk	High-risk	Low-risk	High-risk		Old fit (no population growth)	2000-2019 fit (3.15% population growth)
1	X					$< 10^{-6}$	$< 10^{-6}$
2	X	X				$< 10^{-6}$	$< 10^{-6}$
3	X		X			$< 10^{-6}$	$< 10^{-6}$
4	X			X		0.7071	1
5	X	X	X	X		1	0.0984
6	X				X	$< 10^{-6}$	$< 10^{-6}$
7	X			X	X	0.0010	0.0001
8	X	X	X	X	X	0.0007	$< 10^{-6}$

Table A: Different model structures under consideration and their relative DIC scores for the fitting to 2000–2019 data.

We found that the data most support using Models 4 and 5 when accounting for population growth. This broadly aligns with our previous fit (without population growth) [1] although there is now more support for Model 4 compared to Model 5 using this DIC scoring (a relative DIC of 1 denotes the model with the most support, and the next highest DIC denotes the model with second most support).

Ensemble model approach

Individual model fits (M1–M8) contributed to the ensemble model according to their relative DIC scores. Within 2,000 posteriors in our ensemble model, there are 1837 and 163 unique posteriors from M4 and M5 respectively. Our ensemble results are based on 500 randomly selected posteriors from the ensemble model.

Ensemble posteriors of fitted parameters

Notation	Description	Old fit (no population growth)	2000–2019 fit (3.15% population growth)
R_0	Basic reproduction number (next generation matrix approach)	[1.04, 1.06, 1.10]	[1.05, 1.07, 1.10]
r	Relative bites taken on high-risk humans	[2.97, 5.07, 9.41]	[3.13, 4.43, 7.19]
k_1	Proportion of low-risk, random participating people	[0.65, 0.80, 0.92]	[0.74, 0.82, 0.90]
k_2	Proportion of high-risk, random participating people	[0, 0.01, 0.09]	[0, 0, 0.05]
k_3	Proportion of low-risk, non-participating people	[0, 0.01, 0.18]	[0, 0, 0.05]
k_4	Proportion of high-risk, non-participating people	[0.06, 0.15, 0.27]	[0.09, 0.17, 0.25]
k_A	Relative population of animal reservoirs ($k_A = N_A/N_H$)	[1, 1, 1]	[1, 1, 1]
f_A	Proportion of blood-meals on animal reservoirs	[0, 0, 0]	[0, 0, 0]
η_H^{post}	Treatment rate from stage 1, 1998 onwards (days^{-1})	[9.83, 12.58, 16.27]$\times 10^{-5}$	[12.20, 15.50, 19.59]$\times 10^{-5}$
γ_H^{post}	Exit rate from stage 2 (treatment or death), 1998 onwards (days^{-1})	[4.02, 4.63, 5.31]$\times 10^{-3}$	[3.89, 4.52, 5.23]$\times 10^{-3}$
$b_{\gamma_H^{\text{pre}}}$	Relative exit rate from stage 2 factor, pre-1998	[0.81, 0.95, 1.00]	[0.77, 0.93, 1.00]
γ_H^{pre}	Exit rate from stage 2 (treatment or death), pre-1998 (days^{-1})	[3.59, 4.33, 5.09]$\times 10^{-3}$	[3.34, 4.13, 4.95]$\times 10^{-3}$
Spec	Active screening diagnostic specificity	[0.9991, 0.9993, 0.9995]	[0.9991, 0.9993, 0.9995]
u	Proportion of stage 2 passive cases reported	[0.20, 0.26, 0.32]	[0.25, 0.31, 0.38]
$\eta_{H_{\text{amp}}}$	Relative improvement in passive stage 1 detection rate	[0.18, 1.08, 3.06]	[0.16, 0.86, 2.53]
$\gamma_{H_{\text{amp}}}$	Relative improvement in passive stage 2 detection rate	[0.04, 0.52, 7.67]	[0.01, 0.30, 3.26]
$p_{\text{targetdie}}$	Probability of tsetse hitting a target and dying during host-seeking cycle	[0.2157, 0.4424, 0.6873]	[0.2172, 0.4438, 0.6973]
S1givenFP	Probability of a false positive case would be assigned as stage 1	[0.27, 0.39, 0.54]	[0.25, 0.38, 0.52]

Table B: **Ensemble posteriors of fitted parameters.** Notation, brief description, and [2.5th, 50th, & 97.5th] percentile of posteriors for fitted parameters.

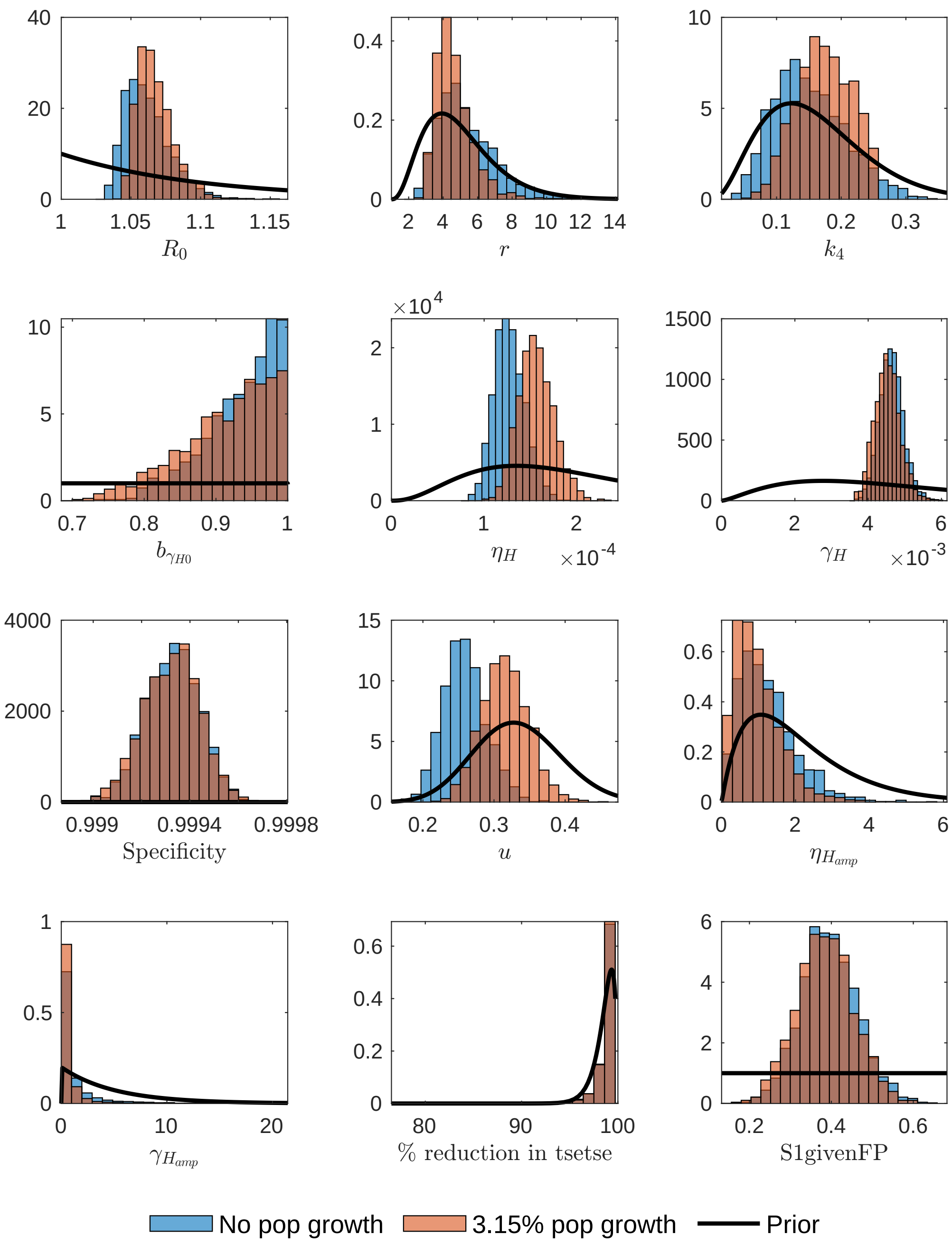

Figure D: **Priors and posteriors for ensemble models fitted to 2000–2019 data for old fit (no population growth) and a new fit (3.15% population growth).** The histograms show the ensemble model posteriors for the two different fits for each of the fitted parameters. The old fit (no population growth) is shown in blue and the 2000–2019 fit (3.15% population growth) is shown in orange. The black curve in each panel shows the prior distribution for that fitted parameter, it represents our belief before fitting was performed.

Ensemble fit

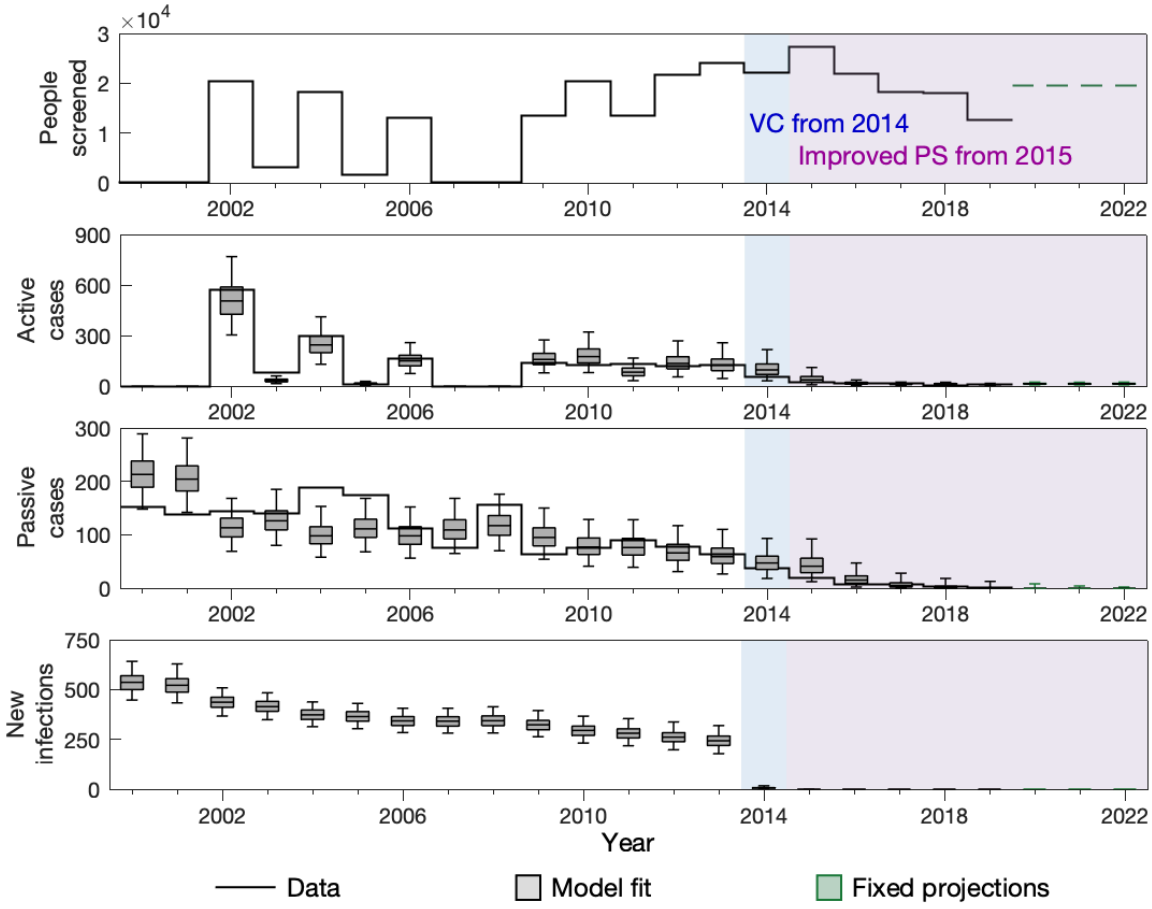

Figure E: **New 2000–2019 ensemble fit (incorporates 3.15% population growth).** The top row shows the number of people screened annually in the Mandoul focus from 2000–2019. Shaded regions denote vector control (VC) starting from 2014 (in blue) and improved passive screening (PS) starting from 2015 (in purple). The second and third rows show the active and passive case data as a solid black line, with grey-filled box and whisker plots denoting the median (centre line), 50% (box edges), and 95% (whiskers) credible intervals for the ensemble fit. Green box and whiskers (2019–2022) show the model projections, updated in the present study to reflect the current known active screening levels. The tsetse reduction from 2014 and the passive detection rate improvement from 2015 were fitted. Inferred new infections each year are shown on the fourth line. This figure is the updated version of Fig S7 in Rock et al. [1], accounting for population growth.

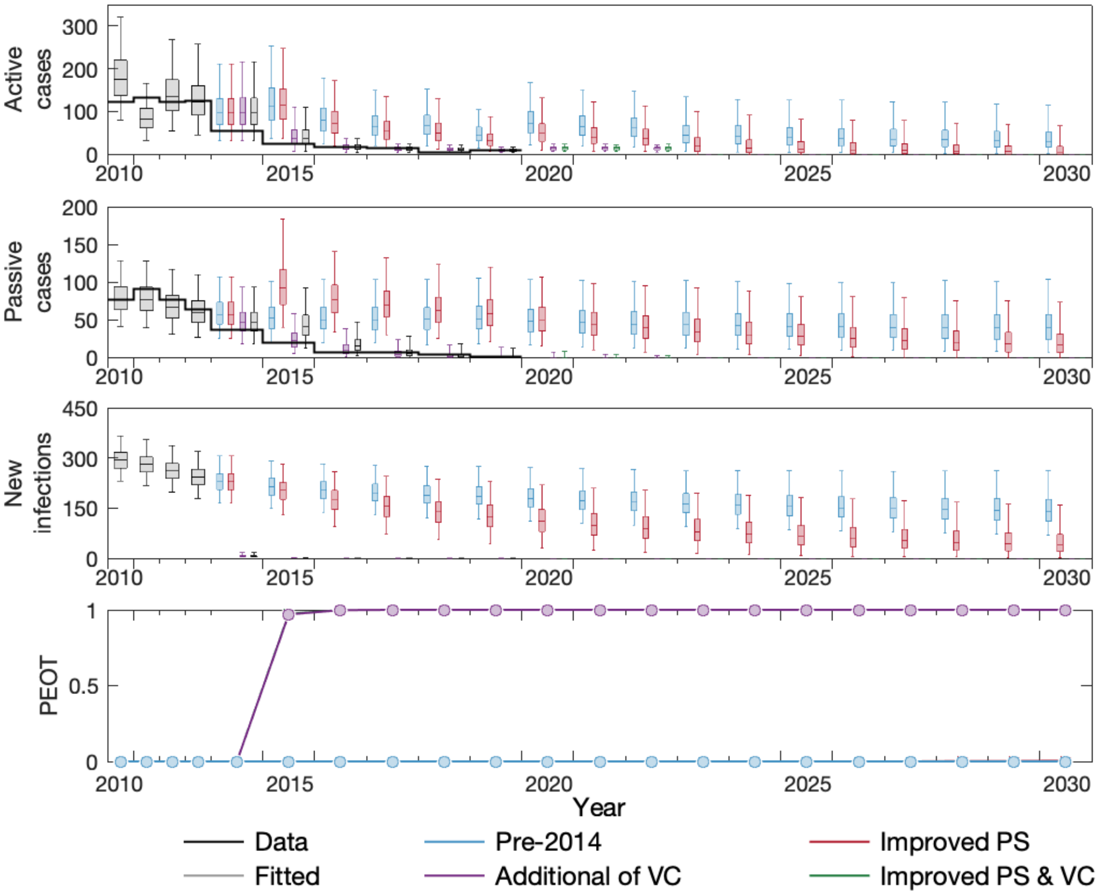

Figure F: **New counterfactual strategy predictions 2014–2030 (incorporates 3.15% population growth).** The first and second rows show the active and passive case data as a solid black line, with grey-filled box and whisker plots denoting the median (centre line), 50% (box edges), and 95% (whiskers) credible intervals for the updated model fit (2000–2019). Counterfactual scenarios (CFSs) are shown from 2014 in other colours. Blue boxes denote the CFS in which no improvements to either vector control (VC) or passive screening (PS) were made, red boxes denote the CFS in which VC was not deployed but enhanced PS was started in 2015, and purple boxes denote the CFS where VC was deployed in 2014, but no enhanced PS was begun in 2015. From 2020 the projections are run under an assumption of mean active screening. The actual strategy switches from grey to green boxes from that year to reflect that it is a projection rather than a fitted. The third row displays the inferred new infections under each scenario, and the last row gives the computed probability of elimination of transmission (EoT) by each year for the different scenarios. This figure is the updated version of Fig S8 in Rock et al. [1], accounting for population growth.

S1.2.3 Passive detection and unreported deaths

In the model, the number of unreported deaths is computed by fitting model parameters to best match model outputs of reported cases via active and passive screening and their stage to the observed case data. Necessarily we have to infer the unreported deaths as there is no data available to inform this in the Mandoul region. Furthermore, most regions impacted by gHAT have limited or no data sources, such as routine autopsies, which could help quantify deaths outside healthcare due to gHAT in other ways.

Fig G shows different ways of leaving the infectious classes in the model (when there is no active detection): (1) detection in stage 1 ($\eta_H(Y)$) or an exit from I_{2Hi} which is either (2) a stage 2 detection (rate $u(Y)\gamma_H(Y)$) OR (3) a death outside of healthcare ($(1-u(Y))\gamma_H(Y)$), where yearly rate $\eta_H(Y)$ and $\gamma_H(Y)$ are given by:

$$\eta_H(Y) = \eta_H^{\text{post}} \left[1 + \frac{\eta_{H_{\text{amp}}}}{1 + \exp\left(-d_{\text{steep}}(Y - d_{\text{change}})\right)} \right] \quad (3)$$

and

$$\gamma_H(Y) = \gamma_H^{\text{post}} \left[1 + \frac{\gamma_{H_{\text{amp}}}}{1 + \exp\left(-d_{\text{steep}}(Y - d_{\text{change}})\right)} \right]. \tag{4}$$

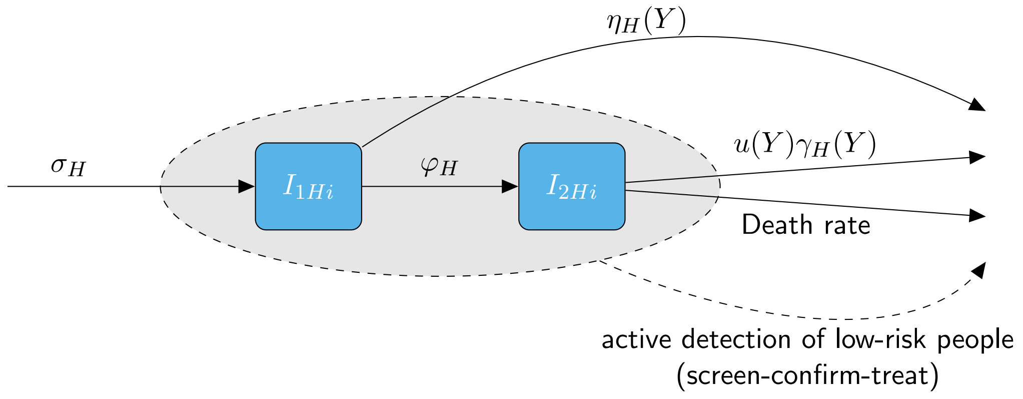

Figure G: **Progression routes for humans once infectious.** The two blue boxes represent people infected in the first and second stages of gHAT respectively. The subscripts denote low-risk ($i = 1, 3$) and high-risk ($i = 2, 4$) sub-populations, some of whom never participate in active screening ($i = 3, 4$). Other disease state compartments are omitted here to focus on passive detection, however, individuals enter from the exposed class "E_{Hi}" at rate σ_H. The gray box denotes individuals who may be actively detected and treated; the coverage of active screening varies from year to year and only people in the "randomly participating" sub-populations may attend ($i = 1, 2$). If an individual is not actively detected, in stage 1 there is a rate of progression to stage 2 (φ_H) and a small rate of detection and treatment ($\eta_H(Y)$) which increases in 2015. Individuals in stage 1 move down each of these pathways proportional to the rates. If not detected actively in stage 2, individuals leave at a rate $\gamma_H(Y)$ which includes disease-induced deaths and passive detection and treatment. The rate of dying remains constant over time and so as the total exit rate $\gamma_H(Y)$ increases after 2015, a higher proportion of stage 2 people are detected and treated ($u(Y)$ becomes larger too).

So at the beginning of 1998 in our simulation, we have $\eta_H(1998) \approx \eta_H^{\text{post}}$ and $\gamma_H(1998) \approx \gamma_H^{\text{post}}$. Fig H show how these rates substantially increase in 2015 when RDTs are introduced in Mandoul by substituting in our fitted model parameters.

We assume that if an infected person had no access to detection or treatment our death rate is the reciprocal of the time until death. When detection and treatment are available our model assumes that the death rate is unchanged (the time to death would still be the same), however, the probability of dying compared to being detected depends on the relative size of the stage 2 detection rate.

$$\text{Death rate (fixed)} = (1 - u(1998))\gamma_H^{\text{post}}. \tag{5}$$

The reporting probability can be written as

$$u(Y) = (1 - \text{Death rate}/\gamma_H(Y)). \tag{6}$$

Without active screening, the probability of infection (any stage) being reported via passive screening is

$$\begin{aligned} \Pr(\text{Passive reporting}) &= \Pr(\text{S1 detection}) + \Pr(\text{S2 detection}) \\ &= \Pr(\text{detection} \mid \text{S1}) + \Pr(\text{S1 progresses to S2}) \times \Pr(\text{detection} \mid \text{S2}) \\ &= \frac{\eta_H(Y)}{\eta_H(Y) + \varphi_H} + \frac{\varphi_H}{\eta_H(Y) + \varphi_H} u(Y). \end{aligned} \tag{7}$$

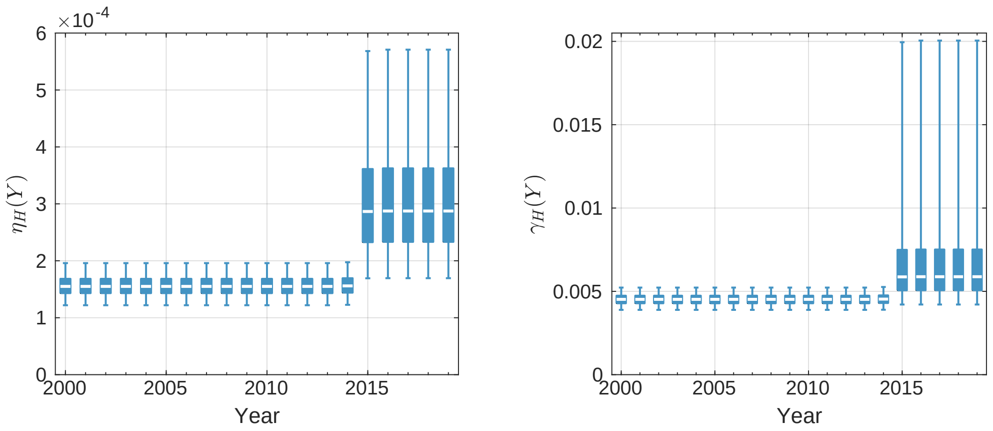

Figure H: **Passive detection rates over time** Left: $\eta_H(Y)$ denotes the rate of detection from the stage 1 compartment (I_{1Hi}). Right: $\gamma_H(Y)$ is the combined rate of exit from the stage 2 compartment (I_{2Hi}), which consists of either detection or death.

Annual passive cases are the total S1 or S2 reports for that year. Annual passive detections from both stages are

$$\text{S1 detection: } P_{M1}(Y) = \int_Y^{Y+1} \eta_H(Y) \sum_i I_{1Hi}(t)\,\mathrm{d}t,$$

$$\text{S2 detection: } P_{M2}(Y) = \int_Y^{Y+1} (\gamma_H(Y) - \text{Death rate}) \sum_i I_{2Hi}\,\mathrm{d}t.$$

We can compute the proportion of all infections reported in the model using:

$$\text{Proportion reported} = 1 - \frac{\text{Deaths}}{\text{Active1} + \text{Active2} + \text{Passive1} + \text{Passive2} + \text{Deaths}}. \tag{8}$$

Using this formula with our simulation results we find that:

- The proportion of infections reported is highly influenced by the amount of AS in the past (Fig I part B in this supplement).
- High variance in our estimates for the proportion of infections reported after 2014 arises due to the introduction of VC and subsequent low numbers of infections (Fig I part A in this supplement).
- PS rates improved in 2015 but lots of potential new infections would have been averted due to the VC starting in 2014. As a result, the proportions reported after 2014 are skewed since these are not new infections and those remaining older infections are more likely to be in people who do not participate in AS (otherwise they would have likely been detected already).

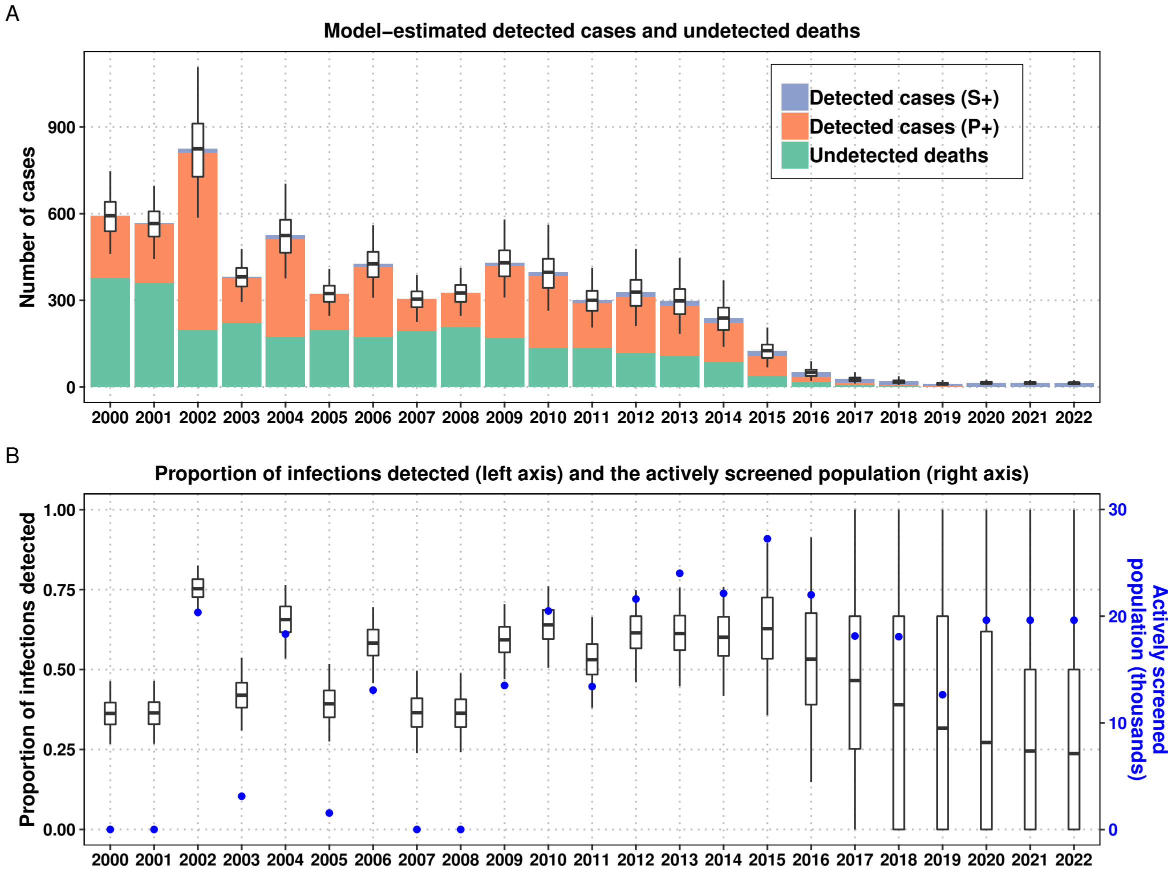

Figure I: Comparison of detected and undetected infections generated by the model and their relationship to active screening intensity. A) detected cases and undetected cases. Box-and-whisker plots show the mean estimate, interquartile range, and 95% confidence interval of the total number of cases. B) the proportion of cases detected and the population tested by mobile screening teams. N.B. 0.5%, 4%, 20%, 44%, 68%, and 83% of 2017–2022 had zero true positive reports and zero deaths in the model simulations, and these simulations are excluded from the proportion calculations in the bottom panel. All results presented in this figure are taken from the model including stochastic sampling.

S1.3 Strategy components

Active screening with traditional vehicle teams and with motorcycle teams (mini teams)

The examination of individuals in their village by mobile teams who screen and confirm cases. Vehicle teams go in two vehicles. Confirmation of suspects is done immediately via parasitology of the blood and cerebrospinal fluid (for those who had trypanosomes in the blood). mAECT (minibar) is very rare, and it has only been used since 2017. See Kohagne *et. al* [6] and Mallaye *et. al* [7] for further explanations of the algorithm for traditional active surveillance.

PNLTHA manages the vehicle teams and they operate on a federal basis, so teams can move across cantonal or health district borders. Multiple villages are screened on the same day. Motorcycle ("mini-mobile") teams are confined to one health district. More recently, motorcycle teams have begun to perform follow-up surveillance (either carrying the parasitology equipment or transporting the suspected case to a hospital with a capacity for parasitology). See the informational brochure by FIND for further explanations of the motorcycle teams [8, 9].

Traditionally, the stage of the disease is determined by microscopy examination of the cerebrospinal fluid, which is extracted via lumbar puncture for cases in which trypanosomes are present in the blood. Depending on whether trypanosomes are present in the cerebrospinal fluid (stage 2) or not (stage 1) patients are referred to the appropriate health center or health district hospital for treatment. In the context of fexinidazole treatment, we do not expect that lumbar punctures will be performed by the active screening team, but rather that patients are referred to a health center that will determine eligibility for fexinidazole treatment. However, fexinidazole has only recently been approved for use and how eligibility takes place and lumbar punctures are administered is to be seen.

Coverage.

The WHO Atlas shows how many activities occurred and how many people were screened, but not how many people were targeted. From programmatic data, we know that in 2017, there were 58 trips to target villages of between 178 to 2520 people. The coverage was an average of 40%, overall; reaching as few as 8% or as many as 79% (IQR: 29%-49%). In the prospective analysis, "Mean AS" is equal to the average number of people screened for the period 2014–2019 (19,628) whereas under a "Max AS" strategy, the number of people screened is the maximum screened in any single year between 2000–2019 (22,146). In the prospective analysis, the actual screening numbers are applied in 2014–2019, then for 2020–2022 "Mean AS" is assumed for all strategies, and for 2023 and beyond "Mean AS", "Max AS", or "Stop AS" is applied depending on the strategy.

Reactive screening

Reactive screening (RS) is equivalent to AS, but it occurs after a case has been identified in the focus where AS had ceased after a three-year period of no cases.

Year	Villages	Pop. Scr.	Cases	Scr per Village
2014	-	22146	-	-
2015	-	17932	6	-
2016	-	11302	5	-
2017	58	9284	0	278
2018	43	10646	0	421
2019	62	11411	10	204
2020	96	18445	11	204

Table C: Summary of active screening activities by traditional teams in trucks. Note: some of these cases might not be parasitologically confirmed.

Passive screening

gHAT screening that occurs in local health posts of patients who present themselves with specific gHAT symptoms. PS, or detection in fixed health facilities is assumed to take place with RDT diagnostic tests used for initial serological screening. For suspects that are RDT-positive, the health worker examines lymph node glands and performs microscopy exams, consisting of a blood sample taken and examined via microscopy,

Year	Villages	Pop. Scr.	Sero+	LTFU	Cases	Scr per Village
2014	0	-	-	-	-	
2015	114	9333	98	missing	6	82
2016	99	10705	176	10	5	108
2017	93	6860	165	95	0	74
2018	101	7437	213	75	0	74
2019	25	1229	53	12	0	49
2020	14	1182	22	1	0	85

Table D: Summary of active screening activities by mini-mobile teams in motorcycles.

and since 2015, via LAMP. For the scale-back, the network of clinics capable of screening patients is scaled back rather than complete cessation; Bodo Hospital hospital, which performs both screening and confirmation capacities would be left.

Coverage. Coverage of PS was assumed to depend on the number of health centers that can perform a serological confirmation of gHAT. See Table E for the data on the coverage throughout 2014–2020.

Year	Facilities - old	Facilities - new	Pop. Scr. Total	Sero+	LTFU	Cases	Scr per Fac.
2014	1		-	-	-	-	
2015	1	9	892	31	5	22	97
2016	1	9	1382	30	0	8	138
2017	1	30	2289	29	0	6	74
2018	1	30	2277	21	7	2	73
2019	1	22	2651	7	3	1	115
2020	1	22	1354	11	6	0	58

Table E: Summary of passive screening in fixed health facilities

Vector control

consists of an annual deployment of Tiny Targets to control the population of tsetse. The impact of vector control in Mandoul is fit by the model; we estimate a 99% decrease in the population of tsetse in the first 4 months.

S1.4 Treatment model

Detected cases (either active or passive) are referred to the district hospital for treatment according to WHO guidelines. The specificity of the screening algorithm as part of the strategy *Mean AS & VC (a)* is 99.93% and for the strategies *Mean AS & VC (b)*, *Mean AS* and *Max AS* it is 100%. *Stop 2023 (No AS or VC)* signifies that AS and VC stop immediately in 2023.

For the retrospective analysis, we built the treatment algorithm according to the practice documented in the literature regarding the use of pentamidine (for S1 cases) and the use of NECT (for stage 2 cases) (see Fig C in this supplement). Some documented cases were assumed to be false positives, since there was evidence of treatment of cases that were confirmed by a CATT 1:16 test. All S1 cases are assumed to have been treated on an outpatient basis. No use of older treatment was assumed (i.e. melarsoprol) in the period of 2014-2020.

For the prospective analysis (2021-2040), we built the treatment algorithm based on the WHO interim recommendations of 2019 [10] regarding the use of fexinidazole, and it is therefore a more elaborate model (see Fig C). For more details, see the supplementary information in Antillon *et. al*, Supplementary Note A, section 2.3 [2].

Patient Characteristic	Parameterization	Summary	Source
Under 6 years old	Beta(152.5, 2427.9)	0.06 (0.05, 0.07)	See S4 Text, Section S4.6.13.
Under 35 kg of weight	Beta(8.3, 359.6)	0.02 (<0.01, 0.04)	See S4 Text, Section S4.6.12.
Late stage-2 disease	Beta(76.9, 44.9)	0.63 (0.55, 0.71)	See S4 Text, Section S4.6.1.

Table F: Parameters for treatment eligibility.

Eligibility	Rationale	Summary
**Stage 1**		
Pentamidine	Under 6 years old (1)	0.06 (0.05, 0.07)
Fexinidazole-inpatient	Over 6 years old but under 35 kg of weight	0.02 (<0.01, 0.04)
Fexinidazole-outpatient	Over 6 years old and over 35 kg of weight	0.92 (0.90, 0.94)
**Stage 2**		
NECT	Under 6 years old or late-stage disease	0.65 (0.57, 0.73)
Fexinidazole-inpatient	Over 6 years old but under 35 kg of weight and early stage-2 disease	<0.01 (<0.01, 0.01)
Fexinidazole-outpatient	Over 6 years old, over 35 kg of weight, and early stage-2 disease	0.34 (0.26, 0.42)

[1] For simplicity, all patients over 6 years old were assumed to be over 20 kg in weight.

Table G: Eligibility for treatment

S1.4.1 Health outcomes denominated as disability-adjusted life-years (DALYs)

As per recommendations of the Bill and Melinda Gates Foundation's reference case and WHO's guidelines for the conduct of cost-effectiveness analyses, we defined the utility of gHAT interventions in terms of disability-adjusted life-years (DALYs) [11–13]. DALYs were discounted at a rate of 3% per year [11, 13]. We follow established conventions to calculate DALYs and evaluate the estimates in present-day terms (after applying the discounting rate) [11, 13, 14]. For the retrospective analysis, costs and DALYs are discounted to present-year values in 2014, the first year of the analysis, and for the prospective analysis present-year values are expressed for the year 2021. For more details, see the supplementary information in Antillon *et. al*, Supplementary Note A, section 3 [2].

S1.5 Cost functions

The total costs are given by following expression:

$$\text{Total costs} = \sum_{i \in \text{all sub-categories}} \left(U_i \times C_i \right)$$

where

i is the cost sub-category of cost (AS, PS, VC, or treatment)

U is the unit of cost, which varies depending on the activity, such as people screened, teams deployed, fixed health centres outfitted with tests, etc.

C is the cost per unit. All costs were denominated in 2020 US$.

U_i or C_i, however, are in turn also functions, as described in the tables for AS (described in section S1.5.1), PS (described in section S1.5.2), VC (described in section S1.5.3), and treatment (described in section S1.5.4).

Treatment	Outcomes	Estimate
**Stage 1**		
Pentamidine	Cured	0.05 (0.05, 0.06)
	Cured with SAEs	<0.01 (<0.01, <0.01)
	Rescue treatment	<0.01 (<0.01, <0.01)
	Death	<0.01 (<0.01, <0.01)
Fexinidazole - inpatient	Cured	0.02 (<0.01, 0.04)
	Cured with SAEs	<0.01 (<0.01, <0.01)
	Rescue treatment	<0.01 (<0.01, <0.01)
	Death	<0.01 (<0.01, <0.01)
Fexinidazole - outpatient	Cured	0.89 (0.87, 0.91)
	Cured with SAEs	0.01 (<0.01, 0.02)
	Rescue treatment	0.02 (<0.01, 0.03)
	Death	<0.01 (<0.01, <0.01)
**All treatments**	**Cured**	**0.97 (0.95, 0.98)**
	**Cured with SAEs**	**0.01 (<0.01, 0.03)**
	**Rescue treatment**	**0.02 (0.01, 0.03)**
	**Death**	**<0.01 (<0.01, <0.01)**
**Stage 2**		
NECT	Cured	0.56 (0.49, 0.64)
	Cured with SAEs	0.06 (0.04, 0.08)
	Rescue treatment	0.03 (0.01, 0.04)
	Death	<0.01 (<0.01, <0.01)
Fexinidazole - inpatient	Cured	<0.01 (<0.01, 0.01)
	Cured with SAEs	<0.01 (<0.01, <0.01)
	Rescue treatment	<0.01 (<0.01, <0.01)
	Death	<0.01 (<0.01, <0.01)
Fexinidazole - outpatient	Cured	0.33 (0.25, 0.41)
	Cured with SAEs	<0.01 (<0.01, <0.01)
	Rescue treatment	<0.01 (<0.01, 0.01)
	Death	<0.01 (<0.01, <0.01)
**All treatments**	**Cured**	**0.90 (0.88, 0.92)**
	**Cured with SAEs**	**0.07 (0.05, 0.09)**
	**Rescue treatment**	**0.03 (0.02, 0.05)**
	**Death**	**<0.01 (<0.01, <0.01)**

Table H: Treatments and outcomes distributions for stage 1 and 2 patients, calculated according to the probability tree in B. SAE: severe adverse events.

S1.5.1 Cost functions: active screening

The yearly costs of AS were calculated as a function of two groups of expenses: 1) overhead costs, and 2) the number of screening tests and confirmation tests that are used across all teams within the health zone. Because no synergies with other disease programs are believed to exist, we have employed a full costing method.

- Overhead costs: overhead costs are split between capital costs and recurrent costs to run an active screening team.
  - Capital costs consist of vehicles, medical equipment, energy (solar panels) and training (which occurs once every few years).
  - Recurrent costs consist of management and consumables that are spent on the team: fuel, staff time, etc.
- Costs that scale by population screened include:
  - CATT tests are scaled up according to the number of people that are screened per year, and a slight mark-up is included to account for wastage of CATT tests.
  - Confirmation tests are counted for all of those who are positive according to the CATT test, both the false positives (which are modelled according to the specificity of the test) and the true positives, which are the outputs of the dynamic model.

– Lumbar punctures are not depicted as part of the AS costs, but are included as part of the treatment costs. Because many patients are eligible for fexinidazole treatment, which does not require lumbar punctures, we include lumbar puncture costs in the treatment portion of the analysis for those patients that are not eligible for fexinidazole. See section S1.5.4.

**Item**	**Units (U)**	**Cost (C)**
Capital (traditional team)	Proportion of patients handled by traditional team	AS capital (traditional team)
Capital (motorcycle team)	Proportion of patients handled by mini-team	AS capital (mini-team)
Management/recurrent expenses (traditional team)	Proportion of patients handled by traditional team	AS recurrent (traditional team)
Management/recurrent expenses (traditional team)	Proportion of patients handled by mini-team	AS recurrent (mini-team)
CATT testing (See Note 1)	AS coverage per year (traditional) × (1+wastage factors for CATT in AS context)	CATT × (1+delivery mark-up)
RDT testing (See Note 1)	AS coverage per year (traditional) × (1+wastage factors for RDT)	RDT × (1+delivery mark-up)
Microscopy/confirmation (traditional team)	(1-CATT specificity) × (AS coverage per year × Population)	Microscopy
Microscopy/confirmation (mini-team)	(1-RDT specificity) × AS coverage per year (mini-team) × (1-LTFU mini-teams)	Microscopy

[1] Ideally, CATT tests would be used for active screening and RDT tests would be used for passive screening because of the high wastage of CATT tests in the context of passive screening settings. In this context, RDT is also used for screening by mini-teams (motorcycle teams) as it lightens the load of supplies necessary for motorcycle teams to take.

Table I: Active screening: cost function

Briefly, we describe these parameters here, but they are displayed in more detail in tables A and D.

Variable Name	Parameterization	Summary	Section in Parameter Glossary
AS coverage per year (traditional team)	Fixed	See Table C	See Table C & Section S4.5.3.
AS coverage per year (motorcycle team)	Fixed	See Table D	See Table D & Section S4.5.4.
Wastage factor for CATT administration in AS context	Beta(8, 92)	0.08 (0.03, 0.14)	Section S4.5.15.
Wastage factor for RDT	Beta(1, 99)	<0.01 (<0.01, 0.04)	Section S4.5.17.
CATT specificity	Beta(31, 2)	1.00 (1.00, 1.00)	Section S4.5.9.
RDT specificity	Beta(3886, 24)	0.99 (0.99, 1.00)	Section S4.5.9.
AS capital costs (traditional team)	Gamma(20000, 0.02)	4,000 (3,982, 4,017)	Section S4.8.4.
AS management costs (traditional team)	Gamma(8.475, 2167)	18,392 (8,203, 32,386)	Section S4.8.5.
AS capital costs (motorcycle team)	Gamma(8.475, 277.10)	2,348 (1,045, 4,119)	Section S4.8.1.
AS management costs (motorcycle team)	Gamma(70.05, 91.56)	6,417 (4,995, 7,958)	Section S4.8.3.
AS followup costs (motorcycle team)	Gamma(70.05, 54.93)	3857 (3007-4785)	Section S4.8.2.
Cost of CATT test	Gamma(23, 0.02)	0.46 (0.29, 0.66)	Section S4.8.6.
Cost of RDT test	Gamma(8.475, 0.19)	1.60 (0.70, 2.82)	Section S4.8.10.
Pr. lost-to-follow-up, RDT+ suspect	Beta, alpha = LTFU, beta = Sero+	See Table D	See Table D & Section S4.5.6.
Cost confirmation (microscopy)	Gamma(8.475, 1.27)	10.80 (4.76, 19.44)	Section S4.8.9.
Cost of delivery (markup)	Beta(15, 25)	0.20 (0.15, 0.25)	Section S4.9.6.

Table J: Components of active screening costs

The cost per year, given the number of people screened, is therefore:

Table K: Cost breakdown for active screening activities

Item	Units (U)	Cost per unit (C)	Cost per category
**Retrospective AS, 2014**			
Capital (annualized, traditional)	1	4,000 (3,982, 4,017)	4,000 (3,982, 4,017)
Capital (annualized, mini-team)	0	2,348 (1,045, 4,119)	0
Management (traditional)	1	18,392 (8,203, 32,386)	18,392 (8,203, 32,386)
Management (mini-team)	0	6,417 (4,995, 7,958)	0
Follow-up (mini-team)	1	3,857 (3,007, 4,785)	3,857 (3,007, 4,785)
Microscopy (traditional)	77.43 (46.17, 108.97)	10.80 (4.76, 19.44)	834 (314, 1,676)
Microscopy (mini-team)	0	10.80 (4.76, 19.44)	0
CATT testing (traditional)	23,917 (22,919, 25,313)	0.55 (0.34, 0.80)	13,125 (8,230, 19,158)
RDT testing (mini-team)	0	1.92 (0.84, 3.37)	0
**Subtotal - Traditional**			**36,351 (24,822, 51,053)**
**Subtotal - Mini-team**			**0**
**Total**			**36,351 (24,822, 51,053)**
**Retrospective AS, 2015**			
Capital (annualized, traditional)	0.66	4,000 (3,982, 4,017)	2,640 (2,628, 2,651)

Table K: Cost breakdown for active screening activities *(continued)*

Item	Units (U)	Cost per unit (C)	Cost per category
Capital (annualized, mini-team)	0.34	2,348 (1,045, 4,119)	798 (355, 1,401)
Management (traditional)	0.66	18,392 (8,203, 32,386)	12,139 (5,414, 21,374)
Management (mini-team)	0.34	6,417 (4,995, 7,958)	2,182 (1,698, 2,706)
Follow-up (mini-team)	1	3,857 (3,007, 4,785)	3,857 (3,007, 4,785)
Microscopy (traditional)	62.92 (37.51, 88.55)	10.80 (4.76, 19.44)	678 (255, 1,362)
Microscopy (mini-team)	53.70 (34.97, 76.54)	10.80 (4.76, 19.44)	579 (232, 1,149)
CATT testing (traditional)	19,434 (18,623, 20,568)	0.55 (0.34, 0.80)	10,664 (6,687, 15,567)
RDT testing (mini-team)	9,363 (9,273, 9,595)	1.92 (0.84, 3.37)	18,000 (7,832, 31,629)
**Subtotal - Traditional**			**26,121 (18,147, 36,309)**
**Subtotal - Mini-team**			**21,559 (11,330, 35,113)**
**Total**			**47,680 (33,879, 64,147)**
**Retrospective AS, 2016**			
Capital (annualized, traditional)	0.51	4,000 (3,982, 4,017)	2,040 (2,031, 2,049)
Capital (annualized, mini-team)	0.49	2,348 (1,045, 4,119)	1,151 (512, 2,019)
Management (traditional)	0.51	18,392 (8,203, 32,386)	9,380 (4,184, 16,517)
Management (mini-team)	0.49	6,417 (4,995, 7,958)	3,144 (2,448, 3,900)
Follow-up (mini-team)	1	3,857 (3,007, 4,785)	3,857 (3,007, 4,785)
Microscopy (traditional)	39.24 (23.40, 55.23)	10.80 (4.76, 19.44)	423 (159, 849)
Microscopy (mini-team)	62.44 (40.62, 89.09)	10.80 (4.76, 19.44)	674 (267, 1,335)
CATT testing (traditional)	12,121 (11,615, 12,828)	0.55 (0.34, 0.80)	6,651 (4,171, 9,709)
RDT testing (mini-team)	10,891 (10,786, 11,161)	1.92 (0.84, 3.37)	20,938 (9,111, 36,793)
**Subtotal - Traditional**			**18,494 (12,620, 25,992)**
**Subtotal - Mini-team**			**25,907 (13,987, 41,708)**
**Total**			**44,401 (30,604, 61,632)**
**Retrospective AS, 2017**			
Capital (annualized, traditional)	0.62	4,000 (3,982, 4,017)	2,480 (2,469, 2,490)
Capital (annualized, mini-team)	0.38	2,348 (1,045, 4,119)	892 (397, 1,565)
Management (traditional)	0.62	18,392 (8,203, 32,386)	11,403 (5,086, 20,079)
Management (mini-team)	0.38	6,417 (4,995, 7,958)	2,438 (1,898, 3,024)
Follow-up (mini-team)	1	3,857 (3,007, 4,785)	3,857 (3,007, 4,785)
Microscopy (traditional)	39.33 (23.45, 55.35)	10.80 (4.76, 19.44)	424 (159, 851)
Microscopy (mini-team)	17.97 (11.26, 26.69)	10.80 (4.76, 19.44)	194.13 (75.82, 391.85)
CATT testing (traditional)	12,149 (11,642, 12,858)	0.55 (0.34, 0.80)	6,667 (4,180, 9,731)
RDT testing (mini-team)	6,964 (6,897, 7,136)	1.92 (0.84, 3.37)	13,388 (5,825, 23,525)
**Subtotal - Traditional**			**20,973 (14,025, 29,935)**
**Subtotal - Mini-team**			**16,912 (9,287, 26,978)**
**Total**			**37,886 (26,988, 51,101)**
**Retrospective AS, 2018**			
Capital (annualized, traditional)	0.59	4,000 (3,982, 4,017)	2,360 (2,350, 2,370)
Capital (annualized, mini-team)	0.41	2,348 (1,045, 4,119)	963 (428, 1,689)
Management (traditional)	0.59	18,392 (8,203, 32,386)	10,851 (4,840, 19,107)
Management (mini-team)	0.41	6,417 (4,995, 7,958)	2,631 (2,048, 3,263)
Follow-up (mini-team)	1	3,857 (3,007, 4,785)	3,857 (3,007, 4,785)

Table K: Cost breakdown for active screening activities *(continued)*

Item	Units (U)	Cost per unit (C)	Cost per category
Microscopy (traditional)	37.30 (22.24, 52.50)	10.80 (4.76, 19.44)	402 (151, 807)
Microscopy (mini-team)	29.48 (18.96, 42.71)	10.80 (4.76, 19.44)	318 (128, 633)
CATT testing (traditional)	11,522 (11,041, 12,194)	0.55 (0.34, 0.80)	6,323 (3,965, 9,229)
RDT testing (mini-team)	7,488 (7,416, 7,674)	1.92 (0.84, 3.37)	14,396 (6,264, 25,297)
**Subtotal - Traditional**			**19,936 (13,325, 28,460)**
**Subtotal - Mini-team**			**18,308 (10,100, 29,153)**
**Total**			**38,244 (27,084, 51,778)**
**Retrospective AS, 2019**			
Capital (annualized, traditional)	0.91	4,000 (3,982, 4,017)	3,640 (3,624, 3,655)
Capital (annualized, mini-team)	0.09	2,348 (1,045, 4,119)	211.35 (94.04, 370.75)
Management (traditional)	0.91	18,392 (8,203, 32,386)	16,737 (7,465, 29,471)
Management (mini-team)	0.09	6,417 (4,995, 7,958)	577 (450, 716)
Follow-up (mini-team)	1	3,857 (3,007, 4,785)	3,857 (3,007, 4,785)
Microscopy (traditional)	40.22 (23.98, 56.60)	10.80 (4.76, 19.44)	433 (163, 871)
Microscopy (mini-team)	5.41 (3.39, 7.81)	10.80 (4.76, 19.44)	58.33 (22.77, 116.32)
CATT testing (traditional)	12,422 (11,904, 13,147)	0.55 (0.34, 0.80)	6,817 (4,275, 9,950)
RDT testing (mini-team)	1,149 (1,138, 1,177)	1.92 (0.84, 3.37)	2,209 (961, 3,881)
**Subtotal - Traditional**			**27,627 (17,846, 40,475)**
**Subtotal - Mini-team**			**3,056 (1,790, 4,719)**
**Total**			**30,683 (20,785, 43,685)**
**Retrospective AS, 2020 and later, prospective mean AS, and prospective max AS 2020-2022**			
Capital (annualized, traditional)	0.69	4,000 (3,982, 4,017)	2,760 (2,748, 2,772)
Capital (annualized, mini-team)	0.31	2,348 (1,045, 4,119)	728 (324, 1,277)
Management (traditional)	0.69	18,392 (8,203, 32,386)	12,691 (5,660, 22,346)
Management (mini-team)	0.31	6,417 (4,995, 7,958)	1,989 (1,549, 2,467)
Follow-up (mini-team)	1	3,857 (3,007, 4,785)	3,857 (3,007, 4,785)
Microscopy (traditional)	47.35 (28.23, 66.64)	10.80 (4.76, 19.44)	510 (192, 1,025)
Microscopy (mini-team)	35.65 (22.71, 51.12)	10.80 (4.76, 19.44)	385 (151, 769)
CATT testing (traditional)	14,626 (14,016, 15,480)	0.55 (0.34, 0.80)	8,026 (5,033, 11,716)
RDT testing (mini-team)	6,145 (6,086, 6,298)	1.92 (0.84, 3.37)	11,815 (5,141, 20,761)
**Subtotal - Traditional**			**23,987 (16,195, 34,049)**
**Subtotal - Mini-team**			**14,917 (8,181, 23,850)**
**Total**			**38,903 (28,127, 52,079)**
**Prospective max AS, 2023 and later**			
Capital (annualized, traditional)	0.69	4,000 (3,982, 4,017)	2,760 (2,748, 2,772)
Capital (annualized, mini-team)	0.31	2,348 (1,045, 4,119)	728 (324, 1,277)
Management (traditional)	0.69	18,392 (8,203, 32,386)	12,691 (5,660, 22,346)
Management (mini-team)	0.31	6,417 (4,995, 7,958)	1,989 (1,549, 2,467)
Follow-up (mini-team)	1	3,857 (3,007, 4,785)	3,857 (3,007, 4,785)
Microscopy (traditional)	65.78 (39.22, 92.57)	10.80 (4.76, 19.44)	709 (267, 1,424)
Microscopy (mini-team)	40.10 (23.67, 60.54)	10.80 (4.76, 19.44)	433 (164, 885)
CATT testing (traditional)	20,317 (19,469, 21,503)	0.55 (0.34, 0.80)	11,149 (6,991, 16,275)
RDT testing (mini-team)	8,537 (8,454, 8,748)	1.92 (0.84, 3.37)	16,412 (7,141, 28,839)
**Subtotal - Traditional**			**27,308 (18,972, 37,960)**

Table K: Cost breakdown for active screening activities *(continued)*

Item	Units (U)	Cost per unit (C)	Cost per category
**Subtotal - Mini-team**			**19,562 (10,244, 31,950)**
**Total**			**46,870 (33,609, 62,635)**

S1.5.2 Cost functions: passive screening (screening at fixed health posts)

The yearly costs of PS were calculated as a function of two groups of expenses: 1) overhead costs, and 2) the number of consultations and screening and confirmation tests that are done in the focus.

- The previously-operating screening & diagnostic center (the Catholic Mission Hospital) is part of the default activities. One screening & confirmation center (Bodo District hospital) is part of the enhanced activities. All "screening-only" centers are part of the *Improved* or *enhanced* PS activities.
- Overhead costs: overhead costs are split between capital costs and recurrent costs to equip a health center to perform serological confirmation for HAT.
  - Capital costs consist of medical equipment, energy (e.g. solar panels) and training (which occurs periodically every few years). These costs are scaled by the number of facilities that can perform serological screening.
  - Recurrent costs consist of management at the national level. As of 2020, since only 23 out of the national 54 clinics are in Mandoul, we attributed $23/54 \times \$6000$ (the cost of management nationally) to the focus.
- Costs that scale by population screened include:
  - RDT tests are scaled up according to the number of people that are screened per year, and a slight mark-up is included to account for wastage of tests.
  - Confirmation tests are counted for all of those who are RDT+: both the false positives (which are modelled as a factor equal to the specificity of the test) and the true positives, which are the outputs of the dynamic model.
  - Lumbar punctures are not depicted as part of the passive surveillance diagnosis costs, but are included as part of the treatment costs. Because many patients are eligible for fexinidazole treatment, which does not require lumbar punctures, we include lumbar puncture costs in the treatment portion of the analysis for those patients that are not eligible for fexinidazole. See section S1.5.4
- Loop-mediated Isothermal Amplification (LAMP) was only available at the Bodo hospital and was performed on very patients per year, and due to an absence of cost estimates, no additional costs were imputed for this.
- Full citations and explanations for the parameters will be given in S4 Text.

Item	Units (U)	Cost (C)
Capital (annualized) - screening and confirmation sites	Number of facilities capable of screening and confirmation within the focus	Capital costs (clinic)
Capital (annualized) - screening only sites	Number of facilities capable of screening only within the focus	Capital costs (RDT clinic)
District management	Per district	District management costs
District management - expanded network	Per district	District management costs for expanded network
Consultation - screening and confirmation sites	PS coverage per year per clinic × Clinics in the focus	Consultation cost
Consultation - screening only sites	PS coverage per year per clinic × Clinics in the focus	Consultation cost
CATT testing - screening and confirmation sites	PS coverage per year per clinic × Clinics in the focus × (1+wastage for CATT in PS context)	CATT × (1+delivery mark-up)
RDT testing - screening only sites	PS coverage per year per clinic × Clinics in the focus × (1+wastage for RDT)	RDT × (1+delivery mark-up)
Microscopy/confirmation (suspects first identified in screening and confirmation sites)	(1-CATT specificity) × (PS coverage per year per clinic × Clinics in the focus)	Microscopy
Microscopy/confirmation (suspects first identified in screening-only sites)	(1-RDT specificity) × (1-Pr. LTFU in PS) × (PS coverage per year per clinic × Clinics in the focus)	Microscopy

[1] NA

Table L: Passive screening: cost function

Variable Name	Parameterization	Summary	Section in Parameter Glossary
Population	Fixed	41,000	See Section S4.5.1.
Old facilities (CATT + Micro)	See Table E in this supplement. Prospective: 2.	See Table E in this supplement.	See Table E in this supplement.
New facilities (RDT only)	See Table E in this supplement. Prospective: 22.	See Table E in this supplement	See Table E in this supplement
Coverage per facility	Normal(97.72, 12.2)	92.00 (39.00, 128.00)	See Table E in this supplement & Section S4.5.11
Wastage factor for CATT administration in PS context	Beta(25, 75)	0.25 (0.17, 0.34)	Section S4.5.16
Wastage factor for RDT administration in PS context	Beta(1, 99)	0.01 (<0.01, 0.04)	Section S4.5.17
CATT specificity	Uniform(0.995, 0.998)	1.00 (1.00, 1.00)	Section S4.5.9
RDT specificity	Beta(3886, 24)	0.99 (0.99, 1.00)	See Table E in this supplement & Section S4.5.10
PS capital costs of a facility (CATT + micro)	Gamma(8.475, 216.24)	1842.20 (809.96, 3355.11)	Section S4.8.11
PS capital costs of a facility (RDT only)	Gamma(8.475, 31.97)	269.54 (125.41, 475.76)	Section S4.8.13
PS management costs (per focus, yearly)	Gamma(32.47, 12.18)	394.84 (271.71, 538.89)	Section S4.8.12
Cost of CATT test	Gamma(22.87, 0.02)	0.46 (0.29, 0.66)	Section S4.8.6
Cost of RDT test	Gamma(8.475, 0.19)	1.61 (0.72, 2.91)	Section S4.8.10
Cost of consultation	Gamma(2.48, 0.79)	1.97 (0.31, 5.14)	Section S4.9.2
Pr. lost-to-follow-up, RDT+ suspect	Year-specific Beta distribution, alpha = LTFU, beta = Sero+ in Table E in this supplement. Prospective: Beta(20.74, 102.70).	See Table E in this supplement. Prospective: 0.16 (0.11, 0.24).	See Table E in this supplement & Section S4.5.12
Cost confirmation (microscopy)	Gamma(8.475, 1.27)	10.69 (4.69, 18.74)	Section S4.8.9
Cost of delivery (markup)	Beta(15, 25)	0.20 (0.15, 0.25)	Section S4.9.6

Table M: Components of passive screening costs

The cost per year, given the number of people screened and the number of health centres available for PS, is shown in table N.

Table N: Cost breakdown for passive screening activities

Item	Units (U)	Cost per unit (C)	Cost per category
**Retrospective PS, 2014**			
Capital - full clinic	1	1,822 (811, 3,208)	1,822 (811, 3,208)
Capital - RDT clinic	0	271 (120, 477)	0
Management	1	396 (272, 545)	396 (272, 545)
OP visit - default	100	2 (0, 5)	196.81 (32.21, 505.83)
OP visit - enhanced	0	1.97 (0.32, 5.06)	0
CATT	125 (117, 134)	0.55 (0.35, 0.80)	68.96 (42.90, 100.84)
RDT	0	1.94 (0.87, 3.46)	0
Microscopy for false positives - default	0.35 (0.21, 0.49)	10.64 (4.78, 18.68)	3.74 (1.37, 7.54)
Microscopy for false positives - enhanced	0	10.64 (4.78, 18.68)	0
**Subtotal - default**			**1,379 (817, 2,118)**
**Subtotal - enhanced**			**1,109 (600, 1,809)**
**Total**			**2,488 (1,433, 3,935)**
**Retrospective PS, 2015**			
Capital - full clinic	2	1,822 (811, 3,208)	3,644 (1,622, 6,417)
Capital - RDT clinic	7	271 (120, 477)	1,895 (837, 3,341)
Management	9	396 (272, 545)	3,567 (2,445, 4,908)
OP visit - default	119	2 (0, 5)	234.20 (38.33, 601.94)
OP visit - enhanced	774	1.97 (0.32, 5.06)	1,523 (249, 3,915)
CATT	149 (139, 159)	0.55 (0.35, 0.80)	82.07 (51.05, 120.00)
RDT	782 (774, 803)	1.94 (0.87, 3.46)	1,514 (678, 2,711)
Microscopy for false positives - default	0.42 (0.25, 0.59)	10.64 (4.78, 18.68)	4.45 (1.63, 8.97)
Microscopy for false positives - enhanced	657 (553, 734)	10.64 (4.78, 18.68)	6,987 (3,050, 12,345)
**Subtotal - default**			**2,539 (1,479, 3,983)**
**Subtotal - enhanced**			**16,912 (11,928, 23,058)**
**Total**			**19,451 (14,006, 26,105)**
**Retrospective PS, 2016**			
Capital - full clinic	2	1,822 (811, 3,208)	3,644 (1,622, 6,417)
Capital - RDT clinic	7	271 (120, 477)	1,895 (837, 3,341)
Management	9	396 (272, 545)	3,567 (2,445, 4,908)
OP visit - default	128	2 (0, 5)	251.92 (41.23, 647.46)
OP visit - enhanced	1251	1.97 (0.32, 5.06)	2,462 (403, 6,328)
CATT	160 (150, 171)	0.55 (0.35, 0.80)	88.27 (54.91, 129.07)
RDT	1,264 (1,251, 1,297)	1.94 (0.87, 3.46)	2,446 (1,095, 4,381)
Microscopy for false positives - default	0.45 (0.27, 0.63)	10.64 (4.78, 18.68)	4.79 (1.75, 9.65)
Microscopy for false positives - enhanced	1,211 (1,107, 1,250)	10.64 (4.78, 18.68)	12,888 (5,658, 22,843)
**Subtotal - default**			**2,563 (1,493, 4,008)**
**Subtotal - enhanced**			**24,684 (16,438, 35,280)**
**Total**			**27,247 (18,708, 38,150)**
**Retrospective PS, 2017**			
Capital - full clinic	2	1,822 (811, 3,208)	3,644 (1,622, 6,417)
Capital - RDT clinic	28	271 (120, 477)	7,579 (3,346, 13,364)
Management	30	396 (272, 545)	11,890 (8,148, 16,360)
OP visit - default	101	2 (0, 5)	198.78 (32.53, 510.89)
OP visit - enhanced	2100	1.97 (0.32, 5.06)	4,133 (676, 10,622)
CATT	126 (118, 135)	0.55 (0.35, 0.80)	69.65 (43.33, 101.85)

Table N: Cost breakdown for passive screening activities *(continued)*

Item	Units (U)	Cost per unit (C)	Cost per category
RDT	2,121 (2,101, 2,178)	1.94 (0.87, 3.46)	4,107 (1,839, 7,355)
Microscopy for false positives - default	0.35 (0.21, 0.50)	10.64 (4.78, 18.68)	3.78 (1.38, 7.62)
Microscopy for false positives - enhanced	2,030 (1,847, 2,098)	10.64 (4.78, 18.68)	21,598 (9,529, 38,157)
**Subtotal - default**			**2,491 (1,436, 3,936)**
**Subtotal - enhanced**			**50,732 (35,695, 69,613)**
**Total**			**53,223 (37,957, 72,207)**
**Retrospective PS, 2018**			
Capital - full clinic	2	1,822 (811, 3,208)	3,644 (1,622, 6,417)
Capital - RDT clinic	28	271 (120, 477)	7,579 (3,346, 13,364)
Management	30	396 (272, 545)	11,890 (8,148, 16,360)
OP visit - default	55	2 (0, 5)	108.25 (17.72, 278.21)
OP visit - enhanced	2160	1.97 (0.32, 5.06)	4,251 (696, 10,926)
CATT	68.76 (64.46, 73.63)	0.55 (0.35, 0.80)	37.93 (23.59, 55.46)
RDT	2,182 (2,161, 2,240)	1.94 (0.87, 3.46)	4,224 (1,891, 7,565)
Microscopy for false positives - default	0.19 (0.11, 0.27)	10.64 (4.78, 18.68)	2.06 (0.75, 4.15)
Microscopy for false positives - enhanced	1,445 (994, 1,828)	10.64 (4.78, 18.68)	15,377 (6,362, 28,363)
**Subtotal - default**			**2,367 (1,347, 3,791)**
**Subtotal - enhanced**			**44,746 (32,269, 60,615)**
**Total**			**47,113 (34,469, 63,145)**
**Retrospective PS, 2019**			
Capital - full clinic	2	1,822 (811, 3,208)	3,644 (1,622, 6,417)
Capital - RDT clinic	20	271 (120, 477)	5,413 (2,390, 9,546)
Management	22	396 (272, 545)	8,719 (5,976, 11,997)
OP visit - default	82	2 (0, 5)	161.38 (26.41, 414.78)
OP visit - enhanced	2464	1.97 (0.32, 5.06)	4,849 (794, 12,464)
CATT	102.52 (96.11, 109.78)	0.55 (0.35, 0.80)	56.55 (35.18, 82.69)
RDT	2,489 (2,465, 2,555)	1.94 (0.87, 3.46)	4,818 (2,158, 8,629)
Microscopy for false positives - default	0.29 (0.17, 0.40)	10.64 (4.78, 18.68)	3.07 (1.12, 6.18)
Microscopy for false positives - enhanced	1,421 (556, 2,174)	10.64 (4.78, 18.68)	15,104 (4,628, 31,654)
**Subtotal - default**			**2,439 (1,401, 3,884)**
**Subtotal - enhanced**			**40,330 (26,899, 58,814)**
**Total**			**42,770 (29,030, 61,594)**
**Retrospective PS, 2020 and later**			
Capital - full clinic	2	1,822 (811, 3,208)	3,644 (1,622, 6,417)
Capital - RDT clinic	20	271 (120, 477)	5,413 (2,390, 9,546)
Management	22	396 (272, 545)	8,719 (5,976, 11,997)
OP visit - default	39	2 (0, 5)	76.76 (12.56, 197.27)
OP visit - enhanced	1320	1.97 (0.32, 5.06)	2,598 (425, 6,677)
CATT	48.76 (45.71, 52.21)	0.55 (0.35, 0.80)	26.90 (16.73, 39.33)
RDT	1,333 (1,320, 1,369)	1.94 (0.87, 3.46)	2,581 (1,156, 4,623)
Microscopy for false positives - default	0.14 (0.08, 0.19)	10.64 (4.78, 18.68)	1.46 (0.53, 2.94)
Microscopy for false positives - enhanced	853 (549, 1,121)	10.64 (4.78, 18.68)	9,077 (3,692, 17,178)
**Subtotal - default**			**2,324 (1,308, 3,732)**
**Subtotal - enhanced**			**29,814 (21,620, 40,291)**

Table N: Cost breakdown for passive screening activities *(continued)*

Item	Units (U)	Cost per unit (C)	Cost per category
**Total**			**32,138 (23,594, 42,762)**

S1.5.3 Cost functions: vector control

The costs of vector control activities for a year cost $56,000 according to a micro-costing effort published in [15] in 2016 USD. When we account for inflation and changes in the exchange of the Central African Franc and the US dollar, we estimate that the equivalent in 2020 USD is 61,238. We applied a gamma distribution in which the 95% CI would span half and double the estimate: Gamma(8.475, rate: 8132). The resulting mean is 68,717 and the 95% CI is 29,881-123,615.

S1.5.4 Cost functions: treatment

Item	Units (U)	Cost (C)
Doctor's consult	All patients	Outpatient consult
Staging cases (supplies and time); patients ineligible for fexinidazole treatment (see Notes 1-2).	Patients in both stages of disease ineligible for fexinidazole treatment.	Lumbar puncture cost
Pentamidine (see Notes 1-3).	Cases of stage 1 disease detected with AS or PS × proportion of patients ineligible for fexinidazole.	Pentamidine × (1+delivery mark-up)
Outpatient care for stage 1 with pentamidine (see Notes 1-3).	Cases of stage 1 disease detected with AS or PS × proportion of patients ineligible for fexinidazole × length of treatment for pentamidine.	Outpatient consult
NECT (see Notes 1-3).	Cases of stage 2 disease detected with AS or PS × proportion of patients ineligible for fexinidazole.	NECT × (1+delivery mark-up)
Inpatient care for stage 2 with NECT (see Notes 1-3).	(Cases stage 2 detected with AS or PS) × proportion of patients ineligible for fexinidazole × length of treatment for NECT.	Inpatient cost per day
Fexinidazole (see Notes 1-2).	Patients in both stages of disease eligible for fexinidazole treatment.	Fexinidazole × (1+delivery mark-up)
Inpatient care for either stage 1 or 2 with fexinidazole (see Notes 1-3)	Patients eligible for fexinidazole on an inpatient basis × length of treatment for fexinidazole.	Inpatient cost per day
Outpatient care for either stage 1 or 2 with fexinidazole (see Notes 1-4).	Patients eligible for outpatient treatment.	Outpatient consult
Treatment for severe adverse events	Patients under each treatment who experience severe adverse events	(Outpatient consult + Inpatient cost per day × length of treatment for severe adverse events)
Microscopy confirmation	All S1, S2 confirmed patients	Microscopy

[1] Fexinidazole was only available in Chad after 2020. See WHO treatment recommendations for eligibility for fexinidazole. While some patients are eligible for fexinidazole (over 6 years of age and below the severe threshold of disease) the recommendations stipulate inpatient care for some patients due to low weight.
[2] The proportion of patients eligible for fexinidazole was determined as follows: (1-proportion of patients under 6 years of age) × (1-proportion of patients with signs of late stage 2 disease).
[3] The proportion of patients who had fexinidazole treatment on an inpatient basis was determined by multiplying the equation in note 2 with the proportion of patients who were over 35 kg of weight.
[4] Fexinidazole is currently recommended on an outpatient basis only for some patients and only as a directly-observed therapy, so we have imputed a cost for the daily administration by a village health worker. For simplicity, we have given this the same value as a regular outpatient visit since it constitutes a small portion of all costs.

Table O: Treatment: cost function

We show here the components of the costs per case treated depending on the stage and the treatment. The parameters for the above table are available in section S4.9 and eligibility distributions are described in

table G.

Variable Name	Parameterization	Summary	Section in Parameter Glossary
Lumbar puncture and laboratory exam - cost	Gamma(2.42, 3.66)	9.04 (1.47, 23.70)	Section S4.8.7
Length of treatment: pentamidine (days)	Fixed	7	Section S4.6.2
Length of hospital stay: NECT (days)	Fixed	10	Section S4.6.4
Length of hospital stay fexinidazole for stage 1 or 2 disease (days)	Fixed	10	Section S4.6.3
Length of severe adverse events (days)	Gamma (1.219, 2.377)	2.89 (0.12, 9.72)	Section S4.6.5
Pr. SAE: pentamidine treatment	Beta(1, 499)	0.0026 (0.0002, 0.0079)	Section S4.6.9
Pr. SAE: NECT treatment	Beta(11.6, 226.4)	0.10 (0.07, 0.13)	Section S4.6.10
Pr. SAE: fexinidazole treatment	Beta(3, 261)	0.01 (<0.01, 0.03)	Section S4.6.11
Outpatient consultation - cost	Gamma(2.48, 0.79)	1.97 (0.34, 4.96)	Section S4.9.1
Hospital day - cost	Gamma(5.45,1.76)	9.62 (3.35, 19.20)	Section S4.9.2
Course of pentamidine - cost	Gamma(100, 0.54)	54.09 (43.97, 64.73)	Section S4.9.3
Course of NECT - cost	Gamma(100, 3.6)	360.15 (293.26, 435.16)	Section S4.9.4
Course of fexinidazole - cost	Gamma(100, 2.2)	219.72 (178.39, 265.18)	Section S4.9.5
Delivery mark-up	Beta(15, 25)	0.20 (0.15, 0.25)	Section S4.9.6

Table P: Parameters for treatment costs

	Pentamidine	NECT	Fexinidazole - inpatient	Fexinidazole - outpatient
Staging	9.04 (1.47, 23.70)	9.04 (1.47, 23.70)	0	0
Doctor's consult	19.66 (3.41, 49.63)	1.97 (0.34, 4.96)	1.97 (0.34, 4.96)	19.66 (3.41, 49.63)
Inpatient care	0	96.24 (33.45, 192.01)	96.24 (33.45, 192.01)	0
Medicine & delivery	64.91 (52.49, 78.34)	432.16 (349.68, 523.90)	263.64 (213.35, 318.69)	263.64 (213.35, 318.69)
Treatment for SAE	<0.01 (<0.01, 0.02)	2.97 (0.25, 11.38)	0.34 (0.02, 1.55)	0.34 (0.02, 1.55)
**Total**	**93.61 (69.34, 128.17)**	**542.37 (433.06, 672.20)**	**362.18 (278.16, 470.28)**	**283.31 (228.16, 344.30)**

Table Q: Cost per person for different gHAT treatments. Because these are costs averaged over all patients and SAEs are rare, the average cost per patient for SAE is low.

References for SI Text 1

1. Rock, K. S. *et al.* Update of transmission modelling and projections of gambiense human African trypanosomiasis in the Mandoul focus, Chad. *Infectious Diseases of Poverty* **11,** 1–13. ISSN: 20499957. https://doi.org/10.1186/s40249-022-00934-8 (2022).
2. Antillon, M. *et al.* Cost-effectiveness of sleeping sickness elimination campaigns in five settings of the Democratic Republic of Congo. *Nature Communications* **13,** 1051. ISSN: 2041-1723. https://doi.org/10.1101/2020.08.25.20181982%20https://www.nature.com/articles/s41467-022-28598-w (Dec. 2022).
3. Rock, K. S., Torr, S. J., Lumbala, C. & Keeling, M. J. Quantitative evaluation of the strategy to eliminate human African trypanosomiasis in the Democratic Republic of Congo. *Parasites and Vectors* **8,** 1–13. ISSN: 17563305. http://dx.doi.org/10.1186/s13071-015-1131-8 (2015).
4. Mahamat, M. H. *et al.* Adding tsetse control to medical activities contributes to decreasing transmission of sleeping sickness in the Mandoul focus (Chad). *PLoS Neglected Tropical Diseases* **11,** 1–16. ISSN: 19352735 (2017).
5. Gelman, A. *et al. Bayesian Data Analysis* Third (CRC Press, 2013).
6. Kohagne Tongué, L., Diarra, A., Peka, M. & Louis, F. Rapport Cout-Efficacite d'une Modification Simple de la Strategie Diagnostique Usuelle de la Trypanosomiase Humane Africaine. *Sciences and Medicines in Africa* **1,** 110–114 (2009).
7. Mallaye, P., Kohagne Tongué, L., Ndeledje, N., Louis, F. J. & Mahamat Hassane, H. Transmission concomitante de trypanosomose humaine et animale : le foyer de Mandoul au Tchad. *Revue d'élevage et de médecine vétérinaire des pays tropicaux* **67,** 5. ISSN: 0035-1865 (2014).
8. Foundation for Innovative New Diagnostics (FIND). *Human African Trypanosomiasis (HAT) Project Update: Using novel strategies to accelerate elimination of human African trypanosomiasis* tech. rep. (Foundation for Innovative New Diagnostics (FIND), 2016), 1–4. http://old.finddx.org/wp-content/uploads/2016/03/HAT-Chad-Eng-FINAL_WEB.pdf.
9. Ndung'u, J. M. *et al.* Trypa-no! contributes to the elimination of gambiense human african trypanosomiasis by combining tsetse control with "screen, diagnose and treat" using innovative tools and strategies. *PLoS Neglected Tropical Diseases* **14,** 1–9. ISSN: 19352735 (2020).
10. WHO Department of Control of Neglected Tropical Diseases. *WHO interim guidelines for the treatment of gambiense human African trypanosomiasis* tech. rep. (World Health Organization, Geneva, Switzerland, 2019). https://www.who.int/trypanosomiasis_african/resources/9789241550567/en/.
11. Claxton, K. P. *et al. The Gates Reference Case for Economic Evaluation* tech. rep. April (The Bill and Melinda Gates Foundation, 2014), 1–68. https://pure.york.ac.uk/portal/en/publications/the-gates-reference-case-for-economic-evaluation(2663ebb2-e102-48c1-af2f-3a482a2eaae0)/export.html.
12. Murray, C. J. Quantifying the burden of disease: The technical basis for disability-adjusted life years. *Bulletin of the World Health Organization* **72,** 429–445. ISSN: 00429686 (1994).
13. World Health Organization. *Making Choices in Health: WHO guide to cost-effectiveness analysis* tech. rep. (Geneva, Switzerland, 2003), 318.
14. Rushby, J. A. & Hanson, K. Calculating and presenting disability adjusted life years (DALYs) in cost-effectiveness analysis. *Health Policy and Planning* **16,** 326–331. ISSN: 14602237. https://academic.oup.com/heapol/article-lookup/doi/10.1093/heapol/16.3.326 (Sept. 2001).
15. Rayaisse, J. B. *et al.* Delivering 'tiny targets' in a remote region of southern Chad: A cost analysis of tsetse control in the Mandoul sleeping sickness focus. *Parasites and Vectors* **13,** 1–16. ISSN: 17563305. https://doi.org/10.1186/s13071-020-04286-w (2020).
