# Supplementary material for: Health economic evaluation of strategies to eliminate gambiense human African trypanosomiasis in the Mandoul disease focus of Chad
Source: PLoS Negl Trop Dis. 2023 Jul 27;17(7):e0011396. doi: 10.1371/journal.pntd.0011396 (PMC10409297; doi:10.1371/journal.pntd.0011396)
Supplement: S2 Text — (PDF) [file pntd.0011396.s002.pdf]

Supporting Information

Health economic evaluations of strategies to eliminate *gambiense* human African trypanosomiasis in the Mandoul disease focus of Chad

S2. Glossary of Technical Terms

Box 1: Glossary (adapted from Antillon et. al 2022 [1] under a CC-BY 4.0 license.)

Epidemiology Terms

Intervention Interventions are separate activities to address a health need (e.g. active screening (AS) or vector control (VC)).

Strategy A strategy is a combination of interventions, carried out with a specific coverage, and in parallel. In this paper, we simulate strategies with and without an improvement in PS and with and without VC.

Elimination of transmission (EoT) Globally this is the 2030 goal for gHAT; here we also consider local EoT for health zones. The feasibility of EoT is expressed as a probability equal to the proportion of our simulations in which new infections equal zero before a given year (usually 2030).

Disability-adjusted life-year (DALY) In order to present the burden of disease in one common metric across diseases, DALYs are calculated in cost-effectiveness analyses. This is the sum of the years lived with disability due to the disease and the years of life lost by fatal cases. See section **??**.

Health Economics Terms

Parameter uncertainty Uncertainty in the level of transmission or in the costs of interventions and treatment due to unknown underlying parameters (see supplementary section **??** for an explanation of our parameterization of the health outcomes and cost model).

Willingness-to-pay (WTP) or cost-effectiveness threshold The amount of money that payers would pay to avert one DALY arising from the disease in the analysis (gHAT). No specific threshold is recommended, but a recent analysis shows that the WTP in Chad is between \$30–518 in 2020 USD per DALY averted [2–4].

Incremental cost-effectiveness ratio A ratio of marginal cost for a marginal benefit, calculated as follows:

$$\text{ICER} = \frac{\Delta\text{Costs}}{\Delta\text{DALYs}} = \frac{\text{Costs}_{\text{strategy}} - \text{Costs}_{\text{next best}}}{\text{Effects}_{\text{strategy}} - \text{Effects}_{\text{next best}}}$$

Not that the ICER is not a strategy against the comparator, but a strategy against the *next best* strategy in terms of costs incurred and DALYs averted.

Cost-effective strategy The strategy where the ICER is less than the WTP (or cost-effectiveness threshold). We say that the cost-effective strategy is "conditional" on the WTP.

Dominated strategy A strategy that costs more than another while yielding worse health outcomes as determined by the DALY calculations. This strategy ought not be implemented.

Weakly dominated strategy (or strategies under extended dominance) A strategy in which the ICER is higher that the next more expensive strategy. Unlike a strongly dominated strategy it costs less than the most expensive strategy, but the cost-per-DALY averted is higher. This strategy ought not be implemented.

Net monetary benefit The net benefits (NMB) framework is derived from ICERs, but also takes uncertainty into account.

$$\text{NMB} \mid \text{WTP} : \text{WTP} \times \Delta\text{DALYs} - \Delta\text{Costs}$$

The optimal strategy at a given WTP is the strategy with the highest mean NMB at that value of WTP.

Optimal strategy Analogous to the cost-effective strategy when no uncertainty is assumed, this is the strategy that is recommended by the NMB framework.

References for SI Text 2

1. Antillon, M. *et al.* Cost-effectiveness of sleeping sickness elimination campaigns in five settings of the Democratic Republic of Congo. *Nature Communications* **13,** 1051. ISSN: 2041-1723. https://doi.org/10.1101/2020.08.25.20181982%20https://www.nature.com/articles/s41467-022-28598-w (Dec. 2022).
2. Bertram, M. Y. *et al.* Cost – effectiveness thresholds: pros and cons. *Bull World Health Organization* **94,** 925–930 (2016).
3. Marseille, E., Larson, B., Kazi, D. S., Kahn, J. G. & Rosen, S. Thresholds for the cost–effectiveness of interventions: Alternative approaches. *Bulletin of the World Health Organization* **93,** 118–124. ISSN: 15640604 (2015).
4. Woods, B., Revill, P., Sculpher, M. & Claxton, K. Country-Level Cost-Effectiveness Thresholds: Initial Estimates and the Need for Further Research. *Value in Health* **19,** 929–935. ISSN: 15244733. http://dx.doi.org/10.1016/j.jval.2016.02.017 (2016).
