# Supplementary material for: Health economic evaluation of strategies to eliminate gambiense human African trypanosomiasis in the Mandoul disease focus of Chad
Source: PLoS Negl Trop Dis. 2023 Jul 27;17(7):e0011396. doi: 10.1371/journal.pntd.0011396 (PMC10409297; doi:10.1371/journal.pntd.0011396)
Supplement: S3 Text — Fig A: Disability-adjusted life-years (DALYs) accrued through time for different counterfactual strategies in the retrospective analysis. Table A: Costs spent vs total costs (in millions USD) for retrospective analysis. Means are given along with 95% prediction intervels (PIs). Fig B: Close-up of costs spent vs to spend in retrospective analysis. A) Costs spent by intervention for each strategy in 2014–2020. B) Costs spent vs those to be spent by activity. See table A for total estimates and uncertainty in each period. Fig C: Disability-adjusted life-years (DALYs) accrued through time for different counterfactual strategies in the prospective analysis. Fig D: Uncertainty in cost-effectiveness for prospective strategies. A) Pie graphs depicting the probability that each strategy is optimal. Strategies with the highest mean net monetary benefit are marked with a “P”. B) Cost-effectiveness acceptability curves (CEACs) with the cost-effectiveness acceptability frontier (CEAFs) marked in bold. Fig E: Cost-effectiveness acceptability curves (CEACs) for retrospective strategies. Cost-effectiveness acceptability curves (CEACs) for retrospective strategies, sensitivity analysis with alternative horizons with the cost-effectiveness acceptability frontier (CEAFs) marked in bold. Fig F: Expected value of perfect partial information (EVPPI) for retrospective analysis. Table B: Sensitivity analysis if fexinidazole had been available since 2014. Summary of effects, costs, elimination of transmission (EoT) by 2030, and cost-effectiveness with and without uncertainty. Highlighted lines are the results that changed after simulating treatment with fexinidazole (compare to Table 3). Means are given along with 95% prediction intervals (PIs). YLL: years of life lost (to fatal disease), YLD: years of life lost to disability, DALYs: disability-adjusted life-years, PS: passive screening, AS: active screening, VC: vector control, ICER: incremental cost-effectiveness ratio, WTP: willingness t [file pntd.0011396.s003.pdf]

Supporting Information

Health economic evaluations of strategies to eliminate *gambiense* human African trypanosomiasis in the Mandoul disease focus of Chad

S3. Supplementary Results

Contents

S3.1 Supplemental results for the retrospective analysis . . . S3.2
S3.2 Supplemental results for the prospective analysis . . . S3.4
S3.3 Sensitivity analysis of time horizon and discounting rate (retrospective analysis) . . . S3.6
S3.4 Sensitivity analysis: results if fexinidazole had been available . . . S3.7
S3.5 Parameter-specific uncertainty analysis (retrospective analysis) . . . S3.8

List of Figures

A Disability adjusted life-years (DALYs) accrued through time for different counterfactual strategies in the retrospective analysis. . . . S3.2
B Close-up of costs spent vs to spend in the retrospective analysis . . . S3.3
C Disability adjusted life-years (DALYs) accrued through time for different counterfactual strategies in the prospective analysis. . . . S3.4
D Uncertainty in cost-effectiveness for prospective strategies. . . . S3.5
E Cost-effectiveness acceptability curves (CEACs) for retrospective strategies . . . S3.6
F Expected value of perfect partial information (EVPPI) for retrospective analysis . . . S3.8

List of Tables

A Costs spent vs total costs for retrospective analysis. . . . S3.2
B Sensitivity analysis if fexinidazole had been available since 2014. . . . S3.7

S3.1 Supplemental results for the retrospective analysis

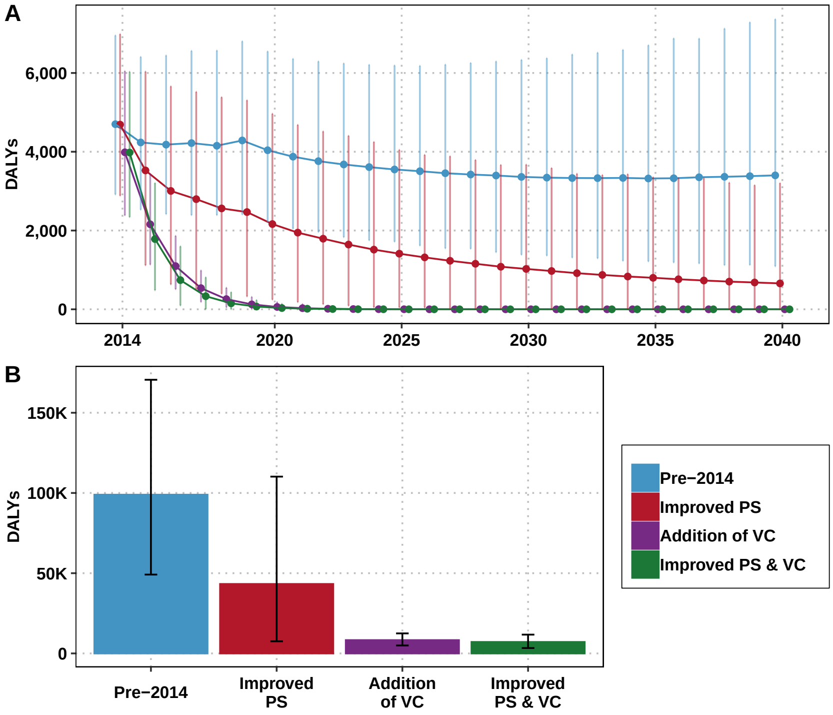

Figure A: Disability adjusted life-years (DALYs) accrued through time for different counterfactual strategies in the retrospective analysis.

Strategies	Costs 2014-2020	Costs 2014-2040	Proportion spent
Pre-2014	0.64 (0.50, 0.81)	2.16 (1.63, 2.83)	0.30 (0.26, 0.34)
Improved PS	0.89 (0.73, 1.09)	2.94 (2.17, 3.72)	0.31 (0.26, 0.37)
Addition of VC	0.91 (0.62, 1.29)	1.25 (0.83, 1.84)	0.73 (0.59, 0.78)
Improved PS & VC	1.15 (0.84, 1.55)	1.82 (1.36, 2.44)	0.63 (0.54, 0.69)

Table A: Costs spent vs total costs (in millions USD) for retrospective analysis. Means are given along with 95% prediction intervels (PIs).

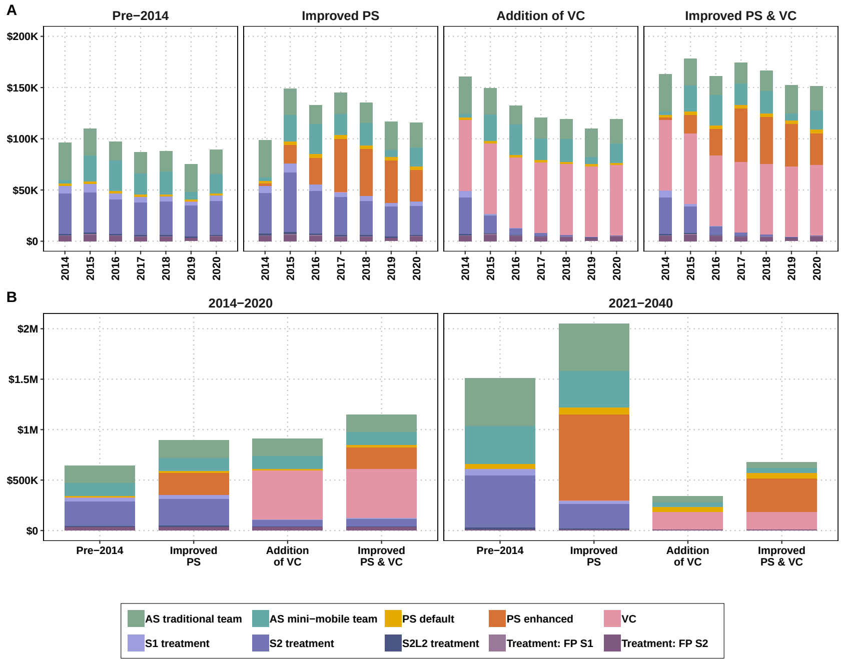

Figure B: Close-up of costs spent vs to spend in the retrospective analysis. A) Costs spent by intervention for each strategy in 2014-2020. B) Costs spent vs those to be spent by activity. See table A for total estimates and uncertainty in each period.

S3.2 Supplemental results for the prospective analysis

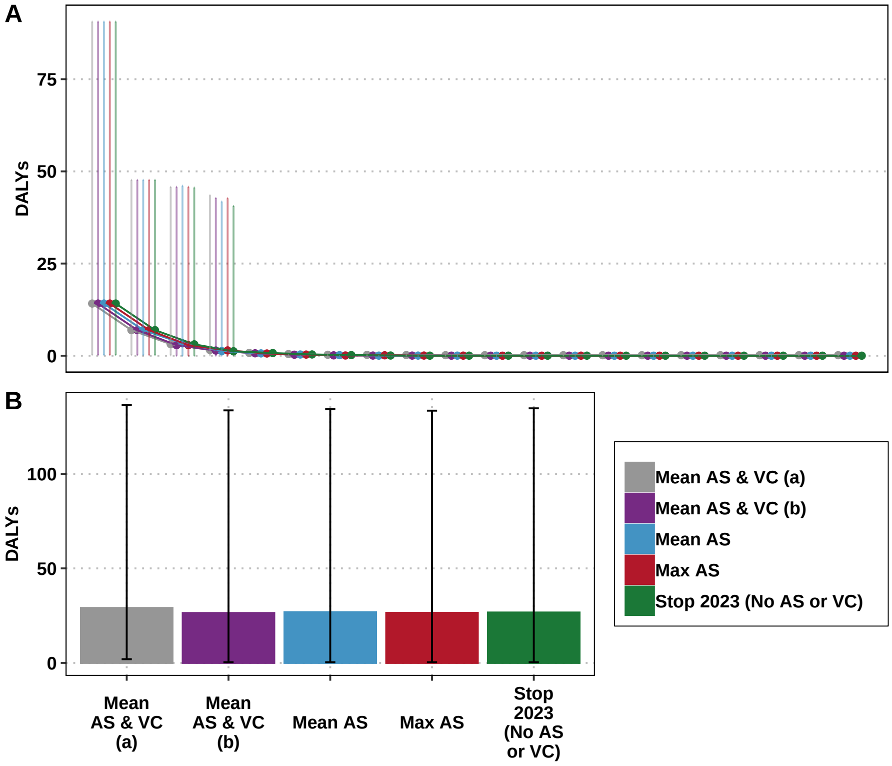

Figure C: Disability adjusted life-years (DALYs) accrued through time for different counterfactual strategies in the prospective analysis.

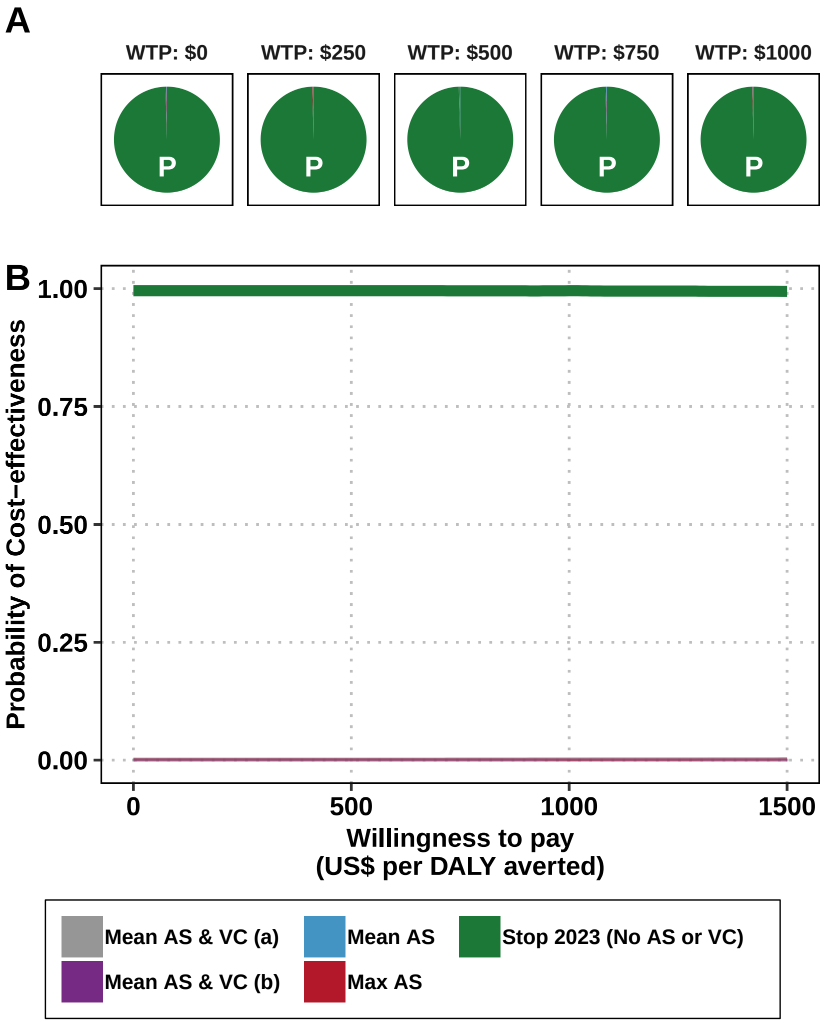

Figure D: Uncertainty in cost-effectiveness for prospective strategies. A) Pie graphs depicting the probability that each strategy is optimal. Strategies with the highest mean net monetary benefit are marked with a "P". B) Cost-effectiveness acceptability curves (CEACs) with the cost-effectiveness acceptability frontier (CEAFs) marked in bold.

S3.3 Sensitivity analysis of time horizon and discounting rate (retrospective analysis)

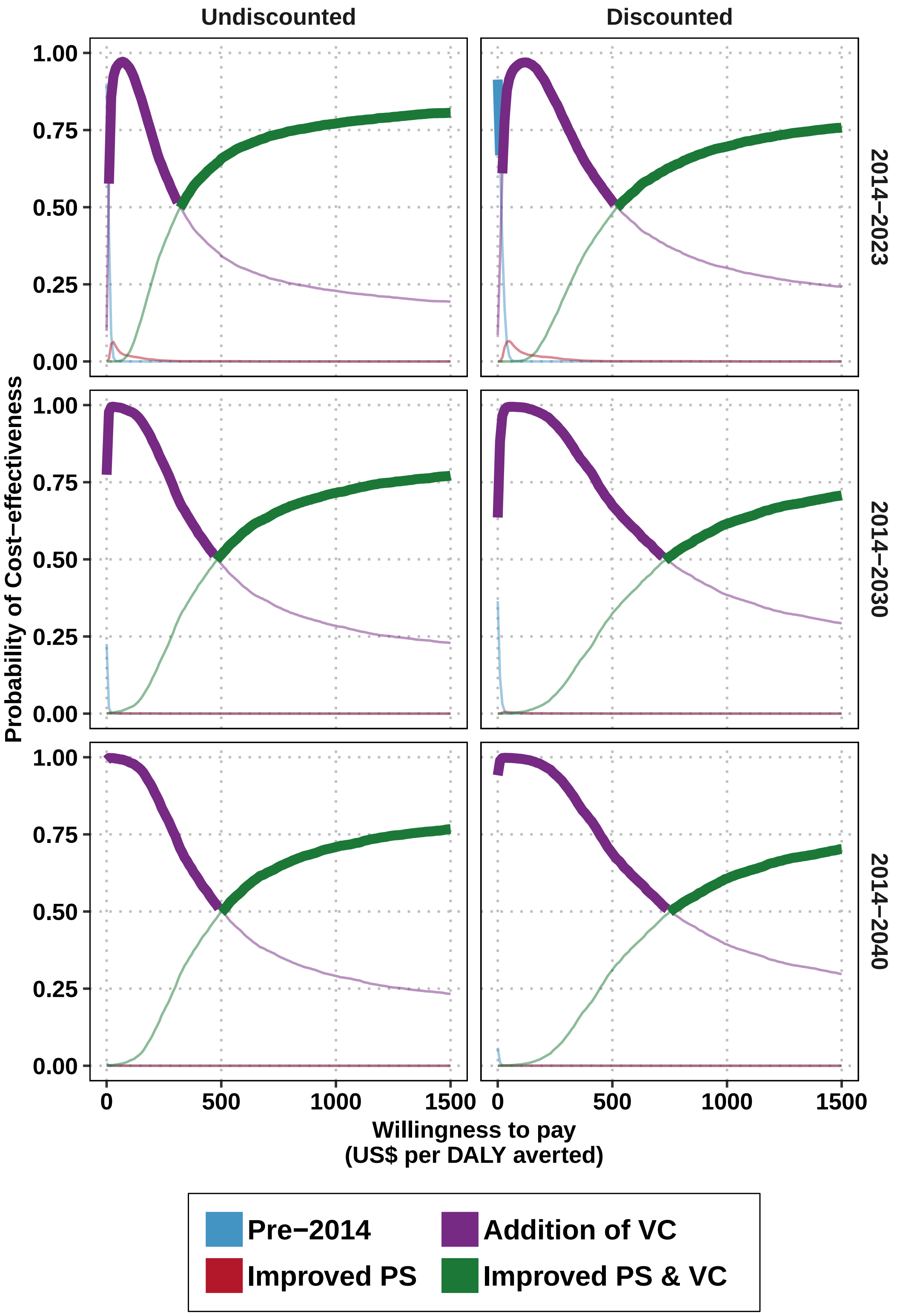

Figure E: Cost-effectiveness acceptability curves (CEACs) for retrospective strategies, sensitivity analysis with alternative horizons with the cost-effectiveness acceptability frontier (CEAFs) marked in bold.

S3.4 Sensitivity analysis: results if fexinidazole had been available

	Pre-2014	Improved PS	Addition of VC	Improved PS & VC
**Health effects**				
Reported cases	2506 (1459, 3956)	1660 (787, 2959)	213 (119, 337)	238 (137, 363)
Deaths undetected	2160 (1062, 3689)	943 (160, 2444)	181 (108, 274)	156 (72, 255)
Cases total	4666 (2564, 7567)	2603 (989, 5341)	394 (253, 564)	394 (248, 569)
Deaths detected[a]	2 (1, 5)	1 (0, 4)	0 (0, 0)	0 (0, 0)
YLD	1707 (972, 2720)	796 (298, 1627)	126 (76, 191)	113 (61, 179)
YLL	97,226 (47,875, 167,451)	42,480 (7,255, 109,979)	8,147 (4,830, 12,355)	7,001 (3,229, 11,652)
DALYs	98,932 (48,985, 169,932)	43,276 (7,700, 111,160)	8,273 (4,959, 12,480)	7,114 (3,340, 11,778)
**Costs, in thousands US$**				
AS costs	1151 (861, 1526)	1131 (808, 1521)	408 (295, 574)	407 (293, 577)
PS costs	66 (38, 105)	1161 (817, 1591)	66 (38, 105)	629 (455, 841)
VC costs	0 (0, 0)	0 (0, 0)	651 (283, 1186)	649 (288, 1179)
Treatment costs	1021 (596, 1624)	697 (346, 1199)	131 (82, 190)	142 (92, 206)
Costs total	2238 (1685, 2925)	2989 (2196, 3785)	1255 (852, 1844)	1826 (1361, 2433)
**EoT**				
Year of EoT	After 2050	2043 (2029, After 2050)	2015 (2015, 2015)	2015 (2015, 2015)
Prob EoT 2030	<0.01	<0.01	>0.99	>0.99
**Cost-effectiveness without uncertainty (discounted)[b]**				
DALYs averted	0	20,718	35,646	36,257
Costs averted	0	547,054	-500,619	-44,237
ICER	Dominated	Dominated	Min Cost	746
**Cost-effectiveness with uncertainty, conditional on WTP[c].**				
WTP: $0	0.04	0	0.96(p)	0
WTP: $250	0	0	0.94(p)	0.06
WTP: $500	0	0	0.69(p)	0.31
WTP: $750	0	0	0.5	0.5(p)
WTP: $1000	0	0	0.39	0.61(p)

[a] Detected deaths are those that occur due to treatment failure of loss-to-follow-up.
[b] Cost-effectiveness results are given for discounted DALYs and costs as per convention
[c] (p) is the preferred strategy; the strategy with the highest mean net monetary benefits

Table B: Sensitivity analysis if fexinidazole had been available since 2014. Summary of effects, costs, elimination of transmission (EoT) by 2030, and cost-effectiveness with and without uncertainty. Highlighted lines are the results that changed after simulating treatment with fexinidazole. Means are given along with 95% prediction intervals (PIs). YLL: years of life lost (to fatal disease), YLD: years of life lost to disability, DALYs: disability-adjusted life-years, PS: passive screening, AS: active screening, VC: vector control, ICER: incremental cost-effectiveness ratio, WTP: willingness to pay (USD per DALY averted), EoT: elimination of transmission.

S3.5 Parameter-specific uncertainty analysis (retrospective analysis)

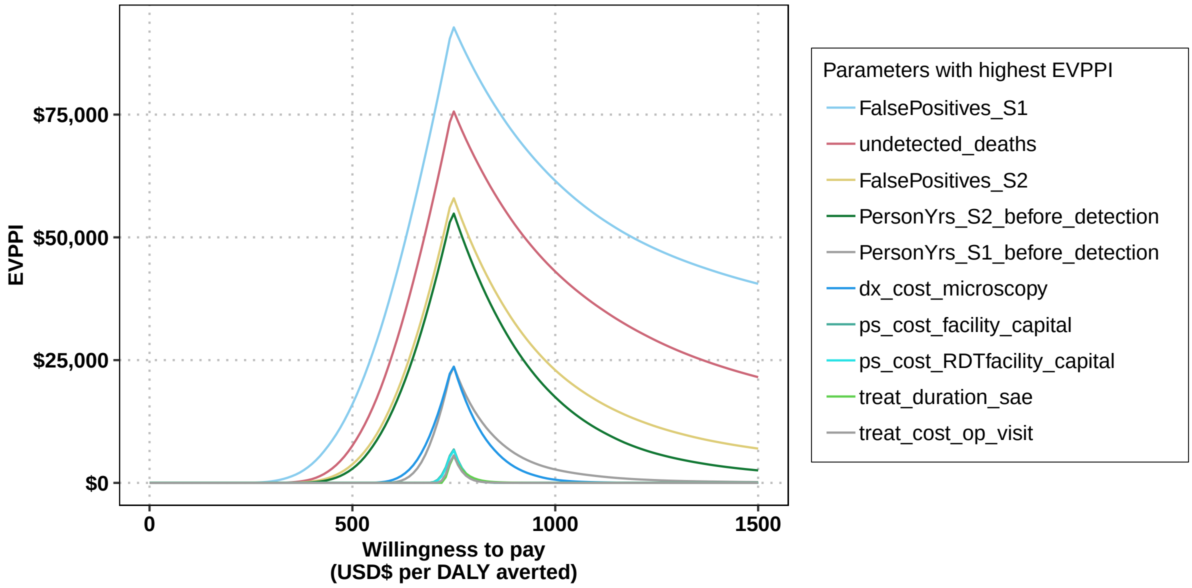

Figure F: Expected value of perfect partial information (EVPPI) for retrospective analysis.
