# Supplementary material for: Health economic evaluation of strategies to eliminate gambiense human African trypanosomiasis in the Mandoul disease focus of Chad
Source: PLoS Negl Trop Dis. 2023 Jul 27;17(7):e0011396. doi: 10.1371/journal.pntd.0011396 (PMC10409297; doi:10.1371/journal.pntd.0011396)
Supplement: Attachment — Submitted filename: Mandoul_Reviewers.docx [file pntd.0011396.s008.docx]

|  | Comment | Response |
| --- | --- | --- |
| 1 | One of my concerns is the fact that the population was kept constant/stable over a 20 years projection period especially given that the analysis focuses on disease elimination. Even with a very low transmission rate, increasing population size may result in more cases than expected under a stable population, especially if population growth is significant in areas/villages of active transmission. Moreover, population increase may affect the number of iterations with resurgence. Though I don't expect it to affect the qualitative nature of the results, it may affect them quantitatively. | "We found this comment an interesting suggestion and we have re-run our simulations with a 3.15% population growth. We have added a supplement to explain the ways in which the model changed due to the addition of population growth and we changed the text to indicate we have assumed population growth. MARINA: change the text and add the supplement." |
| 2 | The second, which is very minor, is for the authors to clarify/provide references for their very rich model parameters. | The full sources and rationale for the parameters are in S4 Text: Parameter Glossary, but we have now also added the citations and the references for each parameter to table 2. |
| 3 | But some Figures and Tables in the main Text are not properly referenced or not referenced at all. For example line 385, Table 3 is referenced but the exact reference seems to be Figure 3. Line 453 references Figure 2B, but it is not clear which figure it is. Line 415, please indicate that Figure A is from Supplementary Information S3. Lines 377 - 379 state that the model has indicated a quick decrease in incidence, but the authors provide no supporting evidence such as Figure. | We have revised the manuscript and made sure the references to figures and tables now match. |

Mandoul CEA reviewers

Reviewer #1

Reviewer #2

|  | Comment | Response |
| --- | --- | --- |
| 1 | Many conclusions are made and then the authors contradict themselves. Some of these conclusions are not a result of the modelling undertaken but authors own suggestions or propositions. I think the paper would be better if the authors simply stick to conclusions based upon their Results of costs and cost effectiveness of the different strategies used in Mandoul to control gHAT. The 21 issues mentioned in Summary and General Comments need to be addressed. | We take this point and have tried to edit our conclusions and discussion sections based on this remark. We do allow ourselves to hypothesise a little in the limitations but try to keep this seperate from conclusions which are a direct result of this study. |
| 2 | ) The results of modelling, however, do not add much new knowledge to what is already known. Conclusions derived are very specific to Mandoul - an area with very low risk of reinvasion making it very difficult to extrapolate conclusions from this particular focus to other foci and caution is needed when drawing broader conclusions from such a very small and peculiar area. They suggest that VC did a very good job with value for money in this area and according to their calculations, the gains that have been made could be maintained by robust passive screening and AS and VC could now be stopped and could be considered for other foci in the country with active transmission (Page 2). This could be possible but in the very next sentence, they dilute their conclusion by saying “Our analysis speaks to comparative efficiency, and it does not take into account all possible considerations; for instance, any cessation of on-going active screening should first consider that strong surveillance activities will be critical to verify elimination of transmission and to protect against the possible importation of infection from neighbouring endemic foci”. Do we need complex modelling to conclude this? | We do not believe that it was at all apparent that the intensive activities performed in Mandoul since 2014 represented a cost-effective use of resources. Our group's previous work did suggest that at least some of the supplementary interventions were necessary to meet elimination with a high degree of certainty (Rock et al, 2022), however with infections targeted for elimination it is not expected that strategies that can achieve this would be efficient. We have added a comment about this in the conclusions.  The second strength of this modelling analysis is it presents estimated costs under a range of plausible future strategies which might be considered by the programme and stresses their expected benefits towards achieving elimination versus economic cost (not just cost/DALY averted). We do not believe it is feasible to make such quantitative conclusions without modelling of this type.  Although we now emphasise more strongly that we can't simple extropolate that these strategies might be cost-effective elsewhere, it does demonstrate that changes to gHAT strategies including possible short-term scale up, have the potential to be cost-effective and this should be explored. Finally it is a limitation that we don't know what WHO's requirement for verification of EoT will be, however we don't think that detracts from our analysis. |
| 3 | "Another example of how the authors water down their own conclusions is provided on Pages 18 &19: Authors conclude that “we believe that the resources devoted to prevention and treatment of gHAT in Mandoul could now be diverted to address the existing burden in Moissala and Maro. Therefore, our study of the cost implications of the activities in Mandoul can serve as an illustrative guide for similar scale-ups of activities where appropriate in the effort to eliminate transmission in other persistently low-incidence locations of Sub-Saharan Africa.” Then on the same page 19 they tear down their own conclusion by saying “Other foci in Chad contain important features different to those in Mandoul including different ecological and epidemiological settings so therefore we cannot conclude that the same strategies that were cost-effective in Mandoul would be cost-effective in Maro and Moissala. For instance, the tsetse population in those regions are not an `ecological island', and Maro is contiguous with the Maitikoulou focus in the Central African Republic, thereby being at higher risk of disease re-introduction. Maro and Moissala likely still need sustained vertical interventions to reach local EoT, but that analysis is beyond the scope of the present study”. " | We have softened the language in the following sentence:  “Therefore, our study of the cost implications of the activities in Mandoul can serve as an illustrative guide for similar scale-ups of activities where appropriate in the effort to eliminate transmission in other persistently low-incidence locations of Sub-Saharan Africa.”  And instead we now say:  "Our study of the cost implications of the activities in Mandoul indicates that initially costly measures aimed at the elimination of transmission in other persistently low-incidence locations of Sub-Saharan Africa could indeed be cost-effective."  Then after the discussion on how Maro and Moissala may be different:  ""However, the results in Mandoul suggest that aggressive strategies aimed at elimination could represent an efficient use of resources.""  The situation in Maro and Moissala is not the same as in Mandoul, however our case-study demonstrates that elimination/expensive interventions can indeed be efficient. As always, analysis in one location must be generalized with caution." |
| 4 | In 2017 (see reference 20), these authors through modelling concluded that a combination of case detection and treatment and vector control may have interrupted transmission and may lead to elimination in the Mandoul focus by 2020. Since then, what is the status in Mandoul? Has elimination been achieved as suggested by modelling at that time? The current conclusions need to be discussed within this context too. | "We have added the following sentence to the introduction (around line 48): ""Rock et al., which utilised 6 more years of case data for model fitting compared to Mahamat et. al, strengthens and reiterates the conclusions that the data are suggestive that the last transmission event occurred in 2015."" There are no establishe procedures by the WHO to ""verify"" the status of any focus, and therefore, we cannot say anything about the status of Mandoul. We have added the following text to clarify: ""The latest, Rock et al. \cite{Rock2022}, using mathematical transmission modeling and data from 2000--2019, strengthens and reiterates the conclusions that the data suggest that the last transmission event occurred in 2015 in Mandoul, although it was expected for the last case to be reported several years after this. Elimination as a public health problem has not yet been validated for Chad and must be done at a country level, rather than focus by focus \cite{Franco2022}. No country has yet been verified as having achieved elimination of transmission and work to define criteria for this goal are not yet defined at time of writing.""" |
| 5 | It is interesting that authors state in page 12 that “… as cases reported would have declined even if Pre-2014 strategy had remained in place, the decline was accelerated by the additional activities of VC deployment and improved PS. Overall, after an initial increase in costs to set up improved PS and to start deployment of VC, costs showed a modest decrease from 2014–2020, but a more accelerated decrease will occur in the next five years, as interruptions in transmission will allow the safe scale-back of vertical activities (Table 3)”. This could be a clear conclusion, in reality there are not so many differences between the different approaches, just when we project in the future. Moreover, this aspect can be biased by the calculation of undetected deaths (too high estimations?). | "We disagree that our results (e.g. those presented in Figure 3) showed that ""there are not so many differences between diffferent approaches"" as the reviewer suggests. Even with the decline in cases and transmission under the Pre-2014 strategy it was very unlikely the 2030 EoT goal would have been met. More over, with our new results accounting for population growth, the difference between the different strategies is even more pronounced. We have tried to modify the text to emphasise the differences much more strongly. The issue of undetected deaths is now addressed in the methods supplementary text 1, Section S1.2.3. " |
| 6 | It also seems clear the importance of maintaining PS in the area after elimination. Maybe it would be most adequate to also keep the reactive screening (perhaps supplemented by reactive VC) more than just ceasing AS. | "Stop 2023 does not have Reactive AS because we wanted to investigate an ""extreme"" lowest-resource scenario. Moreover, we found no evidence that keeping the capacity for reactive screening would be necessary, neither in our conversations with the program nor in the simulations; in only 3 out of 10,000 iterations would reactive screening have been triggered by a parasitological confirmation in passive screening, in effect a 0.03% chance of any additional cases, and we think a 0% chance of triggering ongoing transmission. We have added the following sentence (around line 508): However, active or reactive and passive detection efforts could form part of both control and measurement activities, although we do not believe active or reactive screening is necessary as in all scenarios of the prospective analysis we expect fewer than 8 cases to be detected and for the maintenance of AS to make no difference (Table 4)." |
| 7 | Use of DALYs to measure effectiveness is the usual approach but maybe it is not the best when facing a disease with very low prevalence in the way of elimination. In such a case, one needs to go to the beginning of interventions to estimate all the people non infected because of the application of the strategy of elimination. It requires establishing a starting point that is arbitrary. These authors have considered 2014 as starting point and 2040 as ending point but the situation could be very different if they are considering 2018 as starting point. DALYS are highly influenced by the number of deaths and there could be an overestimation of this factor. | "Arbitrary starting point: We do not believe our starting point was arbitrary; 2014 was the year when intensified activities began, and it is the sensible thing to pick such starting points in the CEA literature. Picking 2018 would have not made sense, as the starting costs would have elapsed and the decision would not have been made in 2018 - 4 years after initiation of new strategies - it would have been made before any initiation. In addition, sensitivity analysis on the horizons, see S3 Text Figure E, show that the horizons make a slight quantitative difference on the threshold of cost-effectiveness, but they would not modify our overall conclusions.  On the other hand, you don't have to go ""all"" the way to the beginning. Consultations with country stakeholders (co-authors on the paper) showed that it would not be sensible to believe that they would have ever not done anything about sleeping sickness cases in 1992. The ""pre-2014"" strategies represented the minimum set of interventions that would have been politically palatable under the circumstances (i.e., security situation and based on WHO guidelines to control gHAT), so there is no need to consider how many lives were saved compared to some theoretical scenario where the disease would have spread unabated.  We agree that for diseases close to elimination of transmission that DALYs averted are often small per dollar spent compared to other diseases such as malaria or helminth infections, however our use of DALYs here does enable us to make this comparitative judgement and, surprisingly, these supplementary new strategies were computed to be likely cost-effective despite the low number of remaining cases. One reason we also present transmission predictions for elimination and total costs is to provide the reader with a sense of the absolute costs to achieve this goal, whether or not it might be cost-effective according to ""traditional"" cost-effectiveness metrics." |
| 8 | The authors consider EoT when 0 cases are reached. This may be different from the WHO recommendation and view of partners that consider at least five years with 0 cases reported. | "We consider EoT when there are no *new* infections to humans. EoT is not 0 new reported cases. Given the long infectious duration of HAT it may be several years between the last transmission event and final case being reported. We agree that, whilst not yet defined, WHO will likely require several years of zero case reporting as an indicator as part of verification of EoT for more than one year.  We realized we needed to be clearer about this, so we changed:  Around line 270: ""The year of elimination of transmission (EoT) is defined in our deterministic model as the first year in which less than one new infection occurs, in line with the proxy threshold used to determine EoT in other gHAT modelling \cite{Huang2022}."" ... It should be noted that this may or may not be equivalent to the year when 0 new cases are detected, and because of the partially observed nature of the new cases, we use detected cases to determine the year of activity cessation.  And we added around line 504:  ""It is critical, however, to factor in data requirements for EoT to be verified, as our metric of elimination -- no new cases -- is only a partially observed process and a relistic metric would have to keep in mind the delay and under-reporting of cases.""" |
| 9 | Page 2: The authors mention “In the first year of renewed reports in Mandoul”. Could they be precise when exactly is that date? | We included the year (1993) to line 9, where the sentence lies. |
| 10 | Page 2: The authors refer to “a painful lumbar puncture for disease staging was required…”. Perhaps it is better to just mention “lumbar puncture…”, as pain is relative. | We have made the suggested change. |
| 11 | Page 3: The global definition of WHO goal for 2020 considers the time. So “High risk areas” should be defined as “more than one new reported case per year per 10,000 people averaged over the previous 5-year period”. The within-country indicator for HAT elimination as a public health problem also includes “over the previous 5-year period”. | We have made the suggested change (line 30-33) |
| 12 | Page 3: When talking about the 2014 previous situation the authors mention “after half a decade of low but unremitting case detection…”, it would be more exact to rephrase as “after a decade of case detection efforts, the number of cases showed a decreasing trend but reaching an unremitting low level of cases in the last years” | We have made the suggested change (line 41) |
| 13 | Page 3: Authors mention “We set out to perform a retrospective analysis for the Mandoul focus in which we look at what was done from 2014 compared to three less ambitious interventions. We examine what would have been the health economic implications if lesser strategies had been performed…”, What are these three less ambitions interventions? Similarly, what do they men by lesser strategies? | "Around line 50 we edited: We set out to perform a retrospective analysis for the Mandoul focus in which we look at what was done from 2014 compared to three less ambitious strategies that would have omitted intervention components of the national programme's strategy. The exact composition of the strategies is in the methods, the whole second subsection and Table 1. We did not consider it appropriate to go into detail on what makes each of the alternative strategies in the introduction, as what is important there is just stating the broad objective of the manuscript." |
| 14 | Page 3: The authors mention that “they tested whether the introduction of fexinidazole in 2014 would have changed that selection”. This test is unclear as fexinidazole was far from being available in 2014 and it came to be used just in 2020. Hence, why to test its introduction in 2014? What are the advantages of fexinidazole with the previous treatment? It has to be considered that before 2014 all the cases were adequately treated. | "We agree that all cases were treated adequately (insofar as most of them recovered completely). Fexinidazole has two small advantages: 1) for cases without signs of advanced stage 2 disease, who are older than 6 years of age, and more than 35 kg in weight (about 40% of cases) there is no need to administer a lumbar puncture for staging before treatment, and 2) the treatment can be given as an outpatient directly-observed therapy for patients over 35kg in weight. For these patients, fexinidazole is non-inferior to NECT and pentamidine in terms of recovery rates. Fexinidazole, in being an outpatient treatment is also a bit cheaper for those patients.  The reason we tested the use of fexinidazole through a sensitivity analysis was to show that even with the hypothetical introduction of fexinidazole, the conclusions of our analysis and its utility as a case study still stand; easier treatment has not made the economic case for expanded screening and vector control any less appealing.  We have changed the wording around this in our introduction and we have added this sentence to the first paragraph of the discussion, to make it clear how that sensitivity analysis fits in with our overall message: Sensitivity analyses showed that even if the decision were to have been taken in the present, with the existence of fexinidazole, which is easier and cheaper to administer, our conclusions remain unchanged." |
| 15 | Page 3: The comparison of Mandoul focus size with the area of Copenhagen or Austin (TX) is unclear. I’m afraid that the comparison is useless for many people as we are not familiar either with the area of Copenhagen and Austin and the use of these standards of size (Austin or Copenhagen) could be misinterpreted. Why not compare with other HAT foci? | We removed the comparison, as we don't know what places will be salient comparisons to the audience. Comparing to other HAT foci is also problematic as they don't always have clear boundaries and for the non-HAT audience, most, if not all, of these places are quite remote and unknown. |
| 16 | Page 4: As far as I know and according to Chad HAT protocols, LAMP is not considered a confirmation test. So the statement “While traditional teams confirmed cases onsite via microscopy exams of blood and cerebrospinal fluid, mini-mobile teams referred RDT+ patients to the two hospitals for confirmation with parasitology or Loop-mediated Isothermal Amplification (LAMP)” should be amended accordingly. The definition of a case in Chad is stated later in page 8. | "We have referred back to the TrypaNO team and the reviewer is right that LAMP is not used for confirmation, but it is used for additional serological screening. From motorcycle teams RDT+ clients have samples taken on filter paper that are referred for follow-up testing by LAMP. Clients that are positive by LAMP are recalled for further testing by microscopy as LAMP is not a confirmatory testing, but its high sensitivity gives additional information on the clinical status of the client. While traditional teams confirmed cases onsite via microscopy exams of blood and cerebrospinal fluid, mini-mobile teams referred RDT+ patients' samples to the two hospitals for additional screening with Loop-mediated Isothermal Amplification (LAMP), then in-person confirmation with parasitology, and treatment." |
| 17 | Page 5 and Fig 1: The authors refer to “less ambitious interventions” applied before 2014, but has the possible impact of some social factors presented in the past as social insecurity and civil war (2005-2010), which was present in the area before 2014 been considered? The AS activities in those years and therefore the number of cases detected was importantly related to the security conditions and this is not mentioned in the paper. This could have had an important impact. For scientists not to familiar reading this paper perhaps authors can clarify what these “less ambitious interventions” are! | "We have modified the article and removed reference to ""less ambitious interventions"" and replaced with ""strategies without supplementary interventions"" . There is no reference to less ambitious interventions in Fig 1.  The strategies are outlined in Table 1 and explained at length in the second section of the methods. For the 2000–2013 period there was very variable active screening in part due to social insecurity and civil war, but also due to variable financing. The activities that were actually implemented in Mandoul for this period are represented all in the model simulations. It is only from 2014 that we simulate the impact of different strategies including the one that was actually conducted (Improved PS & VC) and the counterfactual strategies (including “Pre-2014” which is a continuation of only active and passive screening). We have added the following sentence in the article in line 208, the section of the methods on the transmission model. ”For the 2000–2013 period there was very variable active screening in part due to social insecurity and civil war, but also due to changes in financing. The activities that were actually implemented in Mandoul for this period are represented all in the model simulations”" |
| 18 | Page 6 and Table 1: The case definition (S+ and P+) used in Chad is clarified in page 8 but it should be included before to understand table1. | "This sentence has been added to the end of the caption:  Serological confirmation consists of a person that is positive by CATT 1 in 16 dilution test, and parasitological confirmation is a person whose blood sample contains trypanosomes visible under a microscope." |
| 19 | Page 7: The authors mention that the cases not detected (unreported prevalence) “was assumed to result in undetected deaths”. Maybe this assumption is not correct. Some of these cases will result in undetected deaths but most probably many of them will be detected later when disease is most advanced and showing clear symptoms. This assumption can bias all the calculations done. | "We disagree. We calculate the cases in people that result in undetected death precisely because the disease advances and the person dies. The disease model simulates a continuous process, not a 1-time event, and at any point the infected person can be detected until they die -- so indeed cases not detected are cases never detected. We have changed that line accordingly (line 225) as well as line 234: The key outputs of the transmission and detection model include deaths among infections that were never detected.  The assumptions on deaths are not unfounded, and in fact they are an important output of our modeling. This modeling results in data-driven inference and calculations about 1) the number of infections that must be present to have sustained transmission as long as it was observed, 2) on the ratio of stage 1 and stage 2 disease – providing a snapshot into transmission in years past, 3) the length of time that people can possibly live with the disease (knowing almost all infections would die from the disease without treatment), and 4) the intensity of active screening and the available clinics for passive screening that would have given the infected people an opportunity to be detected, treated, and resolve the disease.  Please see Supplementery Text 1 (supplementary methods), Section S1.2.3 for a new elaboration of this issue." |
| 20 | Page 13: The statement “The improvement of PS would have cut not just a third of cases, demonstrating the impact of PS on transmission dynamics, but more than halved deaths thanks to expanded treatment…” is unclear. How was treatment extended? The extension was mainly to diagnostic capacities through reinforcing PS and AS but the treatment remained the same and was not extended: Either before 2014 or after, “all the cases detected were treated”. Maybe in the model it would be good to know how many cases were detected in the new facilities opened when strategy was reinforced as some data show that cases were mainly detected in the facilities already working before the reinforcement of the activities. It would be also good to know what proportion of new cases were detected by “mini-teams” in the new reinforced approach. Could it be an impact of the improved security situation which allowed a better screening of populations? This should be clarified. | "Within the transmission model we do not explicitly model the number of health facilities with RDT capacity or their individual effectiveness of detecting cases, rather we model an overall improvement for the stage 1 and stage 2 detection rates in the whole focus. We use the fitting process to infer both the 2014 detection rates and also the amount of improvement. Improvements themselves are likely to have arisen both from the increasing in numbers of facilities with RDTs but also the increased training and awareness in all health facilities including those with HAT detection capacity prior to 2015. We're not really concerned with the reason that the screening could be better. It is possible that international organizations were more willing to undertake activities because it was sufficiently safe, but the reason that more screening took place is not important for a model or an analysis like ours. All that matters is that the screening indeed took place. As mentioned in the methods section ""strategy changes in 2014-16"", treatment was also expanded because a second hospital (Bodo District Hospital) was added as a treatment center to the only existing previous center (Catholic Mission Hospital). " |
| 21 | Page 14 and Table 3. The deaths undetected seem too high, probably related to the assumption that the cases detected in a certain moment result in deaths. This can importantly change the estimations. | "See responses to item 7. Our assumptions about unreported deaths are not unfounded, but rather data-driven inference and calculations about 1), the number of cases that must be present to have sustained transmission as long as it was observed, 2) on the ratio of stage 1 and stage 2 disease – providing a snapshot into transmission in years past, 3) the length of time that people can possibly live with the disease (knowing almost all cases die from the disease), and 4) the intensity of active screening and the available clinics for passive screening that would have given the cases an opportunity to be detected, treated, and resolve the disease.  Please see Supplementery Text 1 (supplementary methods), Section S1.2.3 for an elaboration of this issue." |
| 22 | Page 14. It is considered that VC and AS is continued until no more parasitological confirmed cases are detected. Usually, the strategies in use suggest continuing AS during at least three years after the last case is detected. Is this considered by the authors? As stated before “undetected deaths are the most influential parameter when uncertainty analysis. | Yes, this is considered. Please see table 1 under the columns of the stopping criteria where we specify the number of years of zero parasitological (P+) detections required before stopping AS and VC. Yes, the undetected deaths are the most influential parameter. See Section S1.2.3 in Supplementary Text 1 for a discussion of how undetected deaths are estimated. |
| 23 | Page 15. The authors say that the gains obtained could be maintained by robust passive screening. How do they define “Robust” passive screening? | we have removed the word robust |
| 24 | Page 19. While discussing the limitations of the study, the authors mention that “The paper is meant to inform activities, but ultimately, national ownership of the strategies going forward are paramount for the success of the program.” This needs to be clarified- what do they mean by “inform activities”? Secondly isn’t it common knowledge that national ownership of any control strategies is essential for sustainability of the control effort? | Our analysis is informative rather than prescriptive. It is meant to provide suggestions and useful quantitative analysis about plausible strategies. While the optimal strategies are optimal from a cost-effectiveness point of view, we understand that this depends on factors that go beyond the quantitative costs and effects, or the drivers that make activities possible (e.g. the security situation). No, unfortunately it is not obvious that national programs ought to own control strategies. Most of these programs depend on funds from international organizations that provide financing with conditions, and it is our message to these stakeholders that these analysis are meant to be informative but not the only components to be considered at decision-making. |
